# Research on an intelligent drilling parameter optimization method using sliding window segmentation based on the hydraulic-mechanical specific energy model

**DOI:** 10.1371/journal.pone.0339324

**Published:** 2026-01-02

**Authors:** Wei Li, Yapeng Liu, Xiang Li, Bowen Deng, Huan Zhao, Lun Zhu, Ullah Zain

**Affiliations:** 1 The Key Laboratory of Continental Shale Oil and Gas Accumulation and Efficient Development, Northeast Petroleum University, Daqing, Heilongjiang, China; 2 College of Petroleum Engineering, Northeast Petroleum University, Daqing, Heilongjiang, China; 3 National Engineering Research Center of Oil & Gas Drilling and Completion Technology, Daqing, Heilongjiang, China; 4 CNOOC Jiangsu LNG Co., Itd, Yancheng, Jiangsu, China; 5 International Institute of Education, Liaoning Petrochemical University, Fushun, Liaoning, China; Jeddah University: University of Jeddah, SAUDI ARABIA

## Abstract

Optimizing drilling parameters is essential for improving drilling efficiency and reducing operational costs in oil and gas engineering. This study presents an intelligent optimization approach for drilling parameters based on a hydraulic–mechanical specific energy (MSE) model. A time-series data fusion framework integrating Savitzky–Golay filtering, random forest, and hybrid anomaly detection was established to incorporate hydraulic parameters into the MSE model. The model parameters were further refined by coupling the rate of penetration (ROP) equation with a backpropagation (BP) neural network, achieving prediction accuracies of 70% and 90%, respectively. Field validation using 7,231 datasets from four wells revealed that weight on bit, rotary speed, and flow rate are the dominant factors influencing mechanical specific energy. Moreover, the simulated annealing algorithm was employed to globally optimize key parameters, resulting in an average improvement of 43. 34% in drilling efficiency. Compared with conventional MSE-based approaches, the proposed method innovatively integrates sliding window segmentation with the hybrid MSE (HMSE) technique, significantly enhancing time-series data processing. The developed multi-objective optimization model demonstrates superior prediction accuracy and adaptability under field conditions, providing a practical and effective tool for intelligent drilling parameter optimization.

## 1. Introduction

As the petroleum industry advances toward deep and ultra-deep formations, drilling operations increasingly face challenges such as high rock hardness, strong abrasiveness, and complex geological conditions. These factors often result in low ROP, excessive energy consumption, and escalating operational costs. Traditional efficiency-enhancement strategies primarily rely on upgrading drilling tools and modifying equipment, which, although partially effective, are often costly and lack adaptability. In particular, in interbedded formations with alternating soft and hard layers, these approaches frequently lead to abnormal bit wear and excessively high MSE values [1,2]. Therefore, under existing hardware constraints, optimizing drilling parameters to improve efficiency and reduce costs has become a key focus in modern drilling engineering.

Since Teale [3] first introduced the concept of MSE in 1964, it has been widely employed to evaluate the energy efficiency of rock-breaking processes. However, conventional empirical MSE models exhibit limited accuracy and are often inadequate for complex lithological environments. In recent years, data-driven approaches and intelligent algorithms have provided new opportunities for optimizing drilling parameters. For instance, Kunshin et al. [4] employed machine learning to achieve dynamic weight-on-bit optimization; Liu et al. [5] developed a CNN–LSTM model to enhance MSE prediction accuracy; Deng et al. [6] applied a specific energy model to optimize drilling parameters; Song et al. [7] proposed a real-time optimization framework balancing efficiency and cost through multi-objective functions; and Mantegazini et al. [8] identified optimal energy combinations using pre-operational testing and multi-objective optimization. Moreover, Chen et al. [9] integrated LSTM networks with intelligent algorithms for parameter tuning. Qu et al. [10] and Yang et al. [11] respectively developed drilling performance optimization models based on data-driven and multi-objective optimization approaches. Despite these advances, current studies still exhibit notable limitations: (1) most models primarily focus on mechanical parameters while neglecting the systematic influence of hydraulic factors on MSE; (2) existing data preprocessing methods lack robustness against noise and outliers. Furthermore, although ROP prediction models have developed rapidly in recent years—for example, Al-Sahlanee et al. [12] employed ensemble learning methods; Abdulmalek et al. [13] incorporated drilling fluid properties; Ameur-Zaimeche et al. [14] combined ANN and SVM models; Allawi et al. [15] constructed a boosting-based integrated model; and Ansari et al. [16] together with Elkatatny [17] proposed CSVR-ICA and ANN models, respectively—most of these works have not deeply coupled ROP prediction with MSE optimization, thereby limiting their applicability in real drilling scenarios.

**Fig 1:**
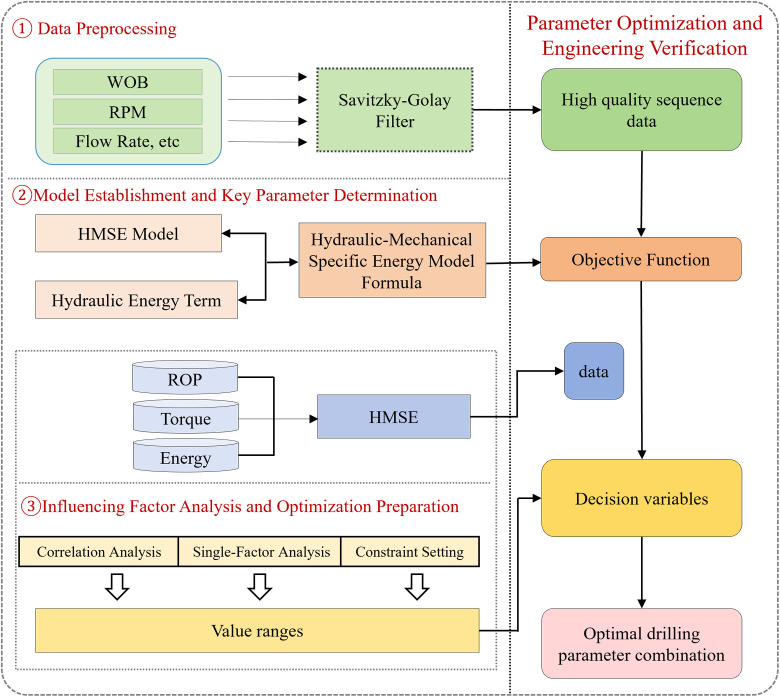
Hydraulic-mechanical specific energy model and optimization flow diagram.

To address these challenges, this study proposes a multi-objective drilling parameter optimization method centered on the MSE concept, incorporating a sliding-window segmentation strategy and a HMSE framework. In the data preprocessing stage, Savitzky–Golay filtering, random forest, and hybrid anomaly detection algorithms are jointly applied to significantly enhance data quality and reliability. At the modeling level, hydraulic parameters are systematically introduced into the MSE framework for the first time, providing a more comprehensive assessment of energy efficiency. In terms of optimization strategy, a simulated annealing algorithm is innovatively integrated to achieve efficient parameter tuning. Validation using a field dataset comprising 7,231 samples from four wells demonstrates that the proposed method substantially improves overall drilling efficiency while maintaining strong robustness. The workflow is illustrated in Fig 1.

## 2. Integrated processing of drilling data

Drilling data constitute a typical continuous time-series signal, susceptible to influences from factors like sensor malfunctions, data transmission errors, storage problems, and human operational errors during data collection, resulting in data anomalies and missing values. Erroneous data can impair the training accuracy of artificial intelligence (AI) models. To tackle this issue, this paper introduces a fusion processing approach for drilling data [18], leveraging Savitzky-Golay filtering, precise missing value imputation, multiple outlier removal techniques, and data smoothing methods.

### 2.1 Basic drilling data acquired

This study employs drilling data collected from the 455m - 465m and 3695m - 3705m intervals of Ultra-deep Well Zhanghai A in the Junggar Basin as the foundational dataset, as presented in Tables 1 and 2 Key issues, including missing values, outliers, and elevated noise levels, are observed within the 457m - 463m and 3700m - 3703m intervals.

**Table 1:** Drilling Data Table for the 455m - 465m Interval of Well Zhanghai A.

Well depth (m)	Drill pressure (kN)	Revolution speed (rpm)	Torque (kN m)	Flow rate (L/s)	Drillability
455	39.06	62.14	7.64	65.43	3.10
456	29.21	62.06	6.26	65.84	2.42
457	0	62.08	5.32	65.84	1.68
458	0	62.46	5.31	65.43	1.68
459	0	62.36	5.49	65.76	1.82
460	0	62.3	5.51	66.49	2.00
461	0	62.72	5.55	65.84	1.91
462	0	62.1	5.55	66.16	2.00
463	0	59.76	2.48	65.33	3.23
464	1.4	59.46	5.17	64.85	1.91
465	49.74	59.2	7.94	63.94	2.75

**Table 2:** Drilling Data Table for the 3695m-3705m Interval of Well Zhanghai A.

Well depth (m)	Drill pressure (kN)	Revolution speed (rpm)	Torque (kN m)	Flow rate (L/s)	Drillability
3695	153.66	78.78	22.47	32.51	3.06
3696	160.18	78.7	23.37	32.18	4.46
3697	165.23	77.62	22.77	32.18	4.48
3698	167.24	78.16	22.98	32.18	5.20
3699	158.7	78.92	24.48	30.54	4.01
3700	121.37	0	0.52	31.2	6.61
3701	126.23	0	0.06	29.87	6.20
3702	216.13	0	0.02	29.87	6.63
3703	223.26	0	0.01	29.87	7.15
3704	119.62	77.66	20.18	30.68	5.41
3705	148.86	79.4	23.67	29.87	4.23

### 2.2 Missing value imputation via random forest

Missing value handling methods can be broadly categorized into two types: indirect imputation and direct deletion. Due to the scarcity of data samples, this study opts for the indirect imputation method to address missing values. Imputation techniques for missing data can be divided into two main categories: value replacement (e. g., mean substitution, hot deck imputation) and value fitting (e. g., regression analysis, maximum likelihood estimation, Random Forest (RF)). This study employs the Random Forest (RF) algorithm, which effectively captures the intricate relationships and nonlinear patterns within and between different intervals of drilling data, as well as the correlations among drilling parameters like weight on bit (WOB), rotational speed, and flow rate, thereby enabling precise estimation of missing values with high robustness [19]. Tables 3 and 4 display the datasets where missing WOB and rotational speed values have been imputed using the Random Forest algorithm, respectively. The Random Forest algorithm exhibits high precision and efficacy in imputing missing values [20].

**Table 3:** Data Table for Imputing Missing Values of Weight on Bit Using Random Forest (455-465m).

Well depth (m)	Drill pressure (kN)	Revolution speed (rpm)	Torque (kN·m)	Flow rate (L/s)	Drillability
455	39.06	62.14	7.64	65.43	3.10
456	29.21	62.06	6.26	65.84	2.42
457	29.66	62.08	5.32	65.84	1.68
458	39.67	62.46	5.31	65.43	1.68
459	49.67	62.36	5.49	65.76	1.82
460	49.68	62.3	5.51	66.49	2.00
461	49.69	62.72	5.55	65.84	1.91
462	49.69	62.1	5.55	66.16	2.00
463	49.70	59.76	2.48	65.33	3.23
464	49.71	59.46	5.17	64.85	1.91
465	49.713	59.2	7.94	63.94	2.75

**Table 4:** Data Table for Imputing Missing Rotary Speed Values Using Random Forest (3695-3705m).

Well depth (m)	Drill pressure (kN)	Revolution speed (rpm)	Torque (kN·m)	Flow rate (L/s)	Drillability
3695	153.66	78.78	22.47	32.51	3.06
3696	160.18	78.70	23.37	32.18	4.46
3697	165.23	77.62	22.77	32.18	4.48
3698	167.24	78.16	22.98	32.18	5.20
3699	158.7	78.92	24.48	30.54	4.01
3700	121.37	78.92	0.52	31.2	6.61
3701	126.23	78.82	0.06	29.87	6.20
3702	216.13	79.12	0.02	29.87	6.63
3703	223.26	78.92	0.01	29.87	7.15
3704	119.62	77.66	20.18	30.68	5.41
3705	148.86	79.40	23.67	29.87	4.23

### 2.3 Hybrid approaches for outlier detection and elimination

Environmental interference and sensor/detection equipment malfunctions at drilling sites result in abnormal data “glitches. “Tables 5 and 6 illustrate a significant data “glitch” at 836m in Well Zhanghai A, characterized by severe data fluctuations in the 3098-3102m depth range. These fluctuations show notable numerical differences and abrupt variations compared to data from neighboring depth intervals. To reduce the impact of these data “glitches” on accuracy, outlier detection and data smoothing must be performed during preprocessing.

**Table 5:** Data Table of Abnormal Weight on Bit for Well Zhanghai A (833-840m).

Well depth (m)	Drill pressure (kN)	Revolution speed (rpm)	Torque (kN m)	Flow rate (L/s)	Drillability
833	126.04	55.64	11.29	59.83	1.82
834	154.05	56.64	13.1	59.83	1.11
835	148.26	55.9	10.72	60.49	1.11
836	4.74	80.44	3.07	61.7	2.19
837	123.42	81.02	10.66	60.06	1.57
838	165.88	80.74	12.54	59.98	1.11
839	166.82	79.32	13.13	61.37	1.26
840	161.64	80.32	13.46	62.03	1.26

**Table 6:** Data Table of Abnormal Weight on Bit for Well Zhanghai A (3095 - 3105m).

Well depth (m)	Drill pressure (kN)	Revolution speed (rpm)	Torque (kN m)	Flow rate (L/s)	Drillability
3095	69.81	82.36	21.38	30.68	4.01
3096	70.17	81.08	20.70	30.68	3.53
3097	77.00	84.14	20.58	31.08	3.62
3098	14.23	84.14	20.11	31.08	3.88
3099	159.98	84.14	20.11	31.08	5.87
3100	208.61	84.14	20.34	31.08	4.81
3101	216.58	84.14	20.58	31.08	4.93
3102	213.23	84.14	20.58	30.68	4.67
3103	84.74	83.22	20.7	30.68	4.42
3104	60.86	82.66	20.61	30.68	3.82
3105	56.78	82.10	21.02	30.68	4.08

**Fig 2:**
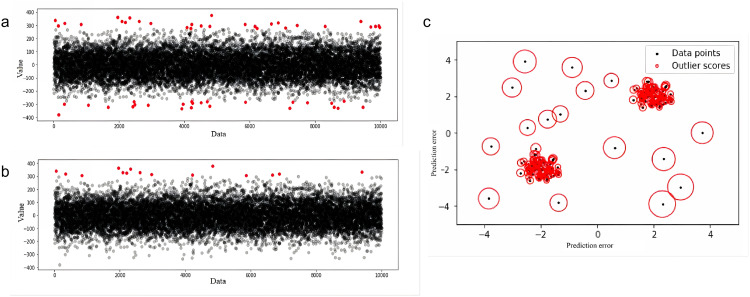
a) Outlier Detection Performance of the 3σ Method; b) Outlier Detection Performance of the K-means Method; c)Outlier Detection Performance of the LOF Method (Black is the standard value, red is the abnormal value).

Common outlier detection methods can be classified into statistical, clustering, and density-based approaches [21,22]. Statistical methods (such as the 3σ criterion) identify outliers by calculating the mean and standard deviation of the data and flagging points that deviate from the mean by more than three standard deviations. This approach is simple and efficient [23,24], as shown in Fig 2(a). Clustering-based methods (such as the K-means algorithm) identify outliers based on the distance between data points and cluster centroids, making them suitable for detecting outliers in global distributions [25], as illustrated in Fig 2(b). Density-based methods (such as Local Outlier Factor, LOF) identify anomalies by comparing the local reachability density of a data point with the density of its neighborhood. These methods effectively detect outliers in high-dimensional data with significantly different local densities [26], as shown in Fig 2(c).

Due to the complex and diverse relationships inherent in drilling data, a single method often struggles to comprehensively detect outliers. Therefore, the study employs an ensemble strategy that normalizes the detection results of the three methods mentioned above, and performs weighted fusion with equal weights (1, 1, 1) to generate a composite outlier score. This approach enhances the robustness and accuracy of the detection [27].

### 2.4 Data fusion and smoothing utilizing the Savitzky-Golay filter

The crux of drilling data preprocessing resides in data fusion and smoothing techniques. Considering the high-dimensional, temporal, and intricate characteristics of drilling data, along with its vulnerability to environmental disruptions and sensor failures, abnormal data, including missing values and outliers, often arise. Such abnormal data can negatively impact subsequent model training and parameter tuning processes. Consequently, it is imperative to adopt effective data fusion and smoothing methodologies to guarantee the precision and dependability of the data. The Savitzky–Golay filter [28] represents a low-pass filtering approach grounded in the least squares principle. It accomplishes smoothing by fitting a low-order polynomial to data points within a localized window, thereby retaining the trend information of the data while mitigating high-frequency noise [29,30]. Tables 7 and 8 showcase the processed data tables following the application of the S-G filter. Notably, the S-G filter effectively smoothed outlier data within the 836m and 3098-3102m depth ranges.

**Table 7:** Data Table After S-G Filter Processing (833 - 840m).

Well depth (m)	Drill pressure (kN)	Revolution speed (rpm)	Torque (kN m)	Flow rate (L/s)	Drillability
833	126.04	55.64	11.29	59.83	1.82
834	154.05	56.64	13.1	59.83	1.11
835	148.26	55.9	10.72	60.49	1.11
836	148.26	80.44	13.14	61.7	1.07
837	123.42	81.02	10.66	60.06	1.57
838	165.88	80.74	12.54	59.98	1.11
839	166.82	79.32	13.13	61.37	1.26
840	161.64	80.32	13.46	62.03	1.26

**Table 8:** Data Table After S-G Filter Processing (3095 - 3105m).

Well depth (m)	Drill pressure (kN)	Revolution speed (rpm)	Torque (kN m)	Flow rate (L/s)	Drillability
3095	69.81	82.36	21.38	30.68	4.01
3096	70.17	81.08	20.70	30.68	3.53
3097	77.00	84.14	20.58	31.08	3.62
3098	69.81	84.14	20.11	31.08	3.88
3099	69.81	84.14	20.11	31.08	5.87
3100	70.17	84.14	20.34	31.08	4.81
3101	69.81	84.14	20.58	31.08	4.93
3102	69.44	84.14	20.58	30.68	4.67
3103	65.22	83.22	20.7	30.68	4.42
3104	60.86	82.66	20.61	30.68	3.82
3105	56.78	82.10	21.02	30.68	4.08

### 2.5 Final result of data processing

To acquire smoothed training data, this section introduces a time-series data fusion and processing approach utilizing Savitzky-Golay filtering. Initially, the random forest algorithm is applied to impute missing values in the drilling dataset. Next, three outlier detection techniques are employed to compute the outlier probabilities for the data. Thereafter, an aggregated outlier probability is derived using a weighted arithmetic mean operator. This probability is then compared against a preset threshold to exclude non-outlier datasets [27]. Ultimately, S-G filtering techniques are implemented to smooth out “glitches” in data fluctuations, yielding more reliable data. To assess the efficacy of the Savitzky-Golay filtering fusion and processing approach, data from four wells in the Zhanghai oilfield are utilized as a case study. Upon processing, a comparative analysis of the original and processed data for the entire well can be conducted. A subset of the data is presented in the Table 9 and 10 below:

**Table 10:** Drilling Parameters After Processing (Part of Well Zhanghai A).

Well depth (m)	Drill pressure (kN)	Revolution speed (rpm)	Torque (kN m)	Flow rate (L/s)	Drillability
64	49.25	54.94	3.48	34.81	1.57
65	49.25	54.96	3.49	34.81	1.57
66	49.25	54.98	3.48	34.81	1.57
67	49.25	54.96	3.42	34.81	1.57
68	48.66	54.94	3.42	34.81	1.57
69	48.07	54.94	3.45	34.81	1.57
70	48.07	54.94	3.45	34.81	1.57
71	45.20	54.94	3.45	34.81	1.44
72	48.07	54.94	3.49	34.81	1.44
73	44.59	54.94	3.45	34.81	1.38
74	44.20	54.94	3.45	34.81	1.38
75	44.2	54.94	3.41	34.81	1.38
76	44.2	54.94	3.31	34.81	1.31
77	44.2	54.94	3.45	34.81	1.31
78	44.2	54.94	3.45	34.81	1.31
79	39.47	54.94	3.45	34.81	1.31
80	42.07	54.94	3.4	34.81	1.31
81	42.07	54.94	3.42	34.81	1.31
82	39.47	54.94	3.42	34.81	1.31
83	39.23	54.94	3.45	34.81	1.31
84	39.23	54.94	3.45	34.81	1.31
85	39.23	54.96	3.33	34.81	1.31
86	39.23	54.96	3.42	34.81	1.31
87	39.23	54.96	3.42	34.81	1.31

**Table 9:** On-site Drilling Parameters (Part of Well Zhanghai A).

Well depth (m)	Drill pressure (kN)	Revolution speed (rpm)	Torque (kN m)	Flow rate (L/s)	Drillability
64	63.37	55.06	4.19	33.8	2.36
65	73.63	54.68	5.09	34.2	1.68
66	44.98	54.98	3.48	35.1	1.82
67	35.70	54.96	3.10	34.8	1.26
68	35.96	54.84	3.18	34.5	1.71
69	54.97	54.24	3.99	34.5	1.57
70	50.44	54.78	3.80	34.8	1.57
71	57.30	54.62	4.31	34.5	2.29
72	48.07	54.94	3.49	34.8	1.82
73	57.28	54.70	3.77	34.8	1.82
74	37.51	55.02	3.00	34.8	1.68
75	39.47	54.96	3.41	34.8	1.11
76	44.20	54.96	3.31	34.8	1.57
77	57.79	55.06	3.94	34.2	1.11
78	80.02	55.24	5.28	33.8	0.94
79	65.87	55.36	4.58	34.8	0.94
80	45.43	54.94	3.40	35.1	1.44
81	37.71	54.80	2.90	34.8	1.26
82	38.53	54.62	3.00	34.8	1.11
83	39.23	54.64	3.11	34.8	1.11
84	47.75	54.66	3.33	34.81	0.67
85	42.02	54.56	3.13	34.81	0.44
86	42.07	54.8	3.02	35.14	4.29
87	40.71	54.9	2.79	34.81	2.56

**Fig 3:**
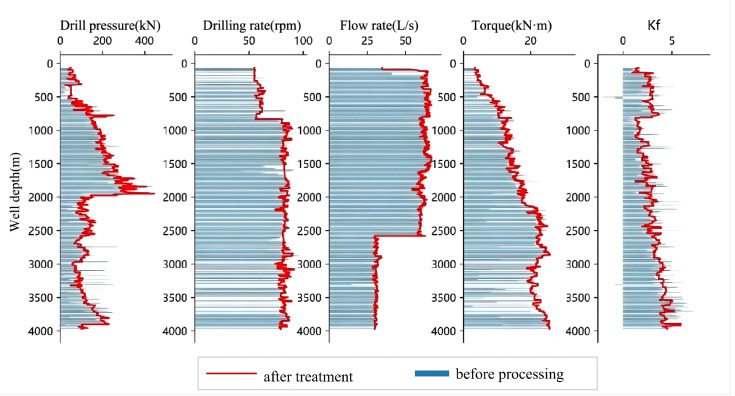
Comparison of Raw Data and Processed Data for Well Zhanghai A.

**Fig 4:**
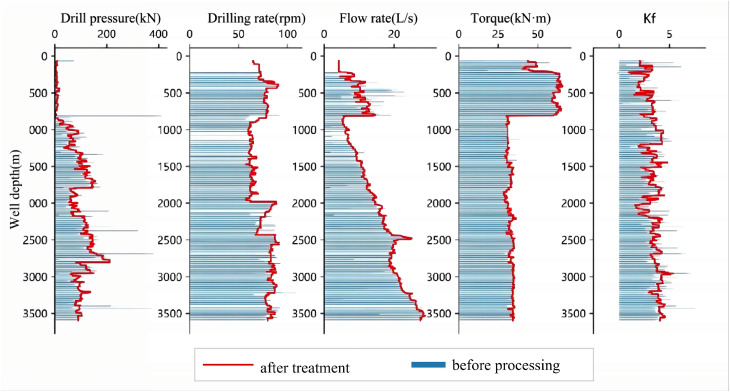
Comparison of Raw Data and Processed Data from Well Zhanghai B.

**Fig 5:**
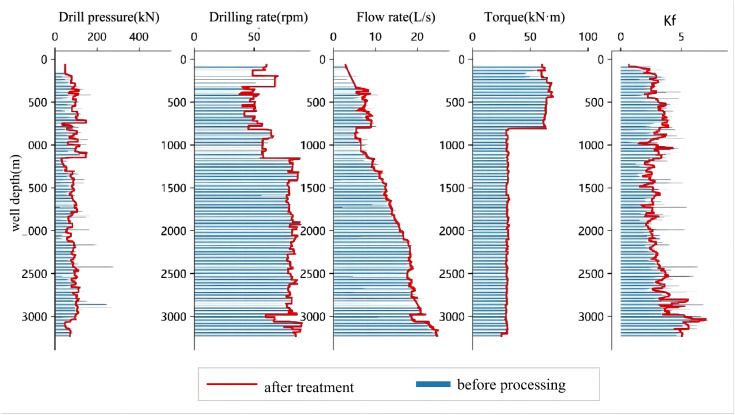
Comparison of Raw Data and Processed Data from Well Zhanghai C.

**Fig 6:**
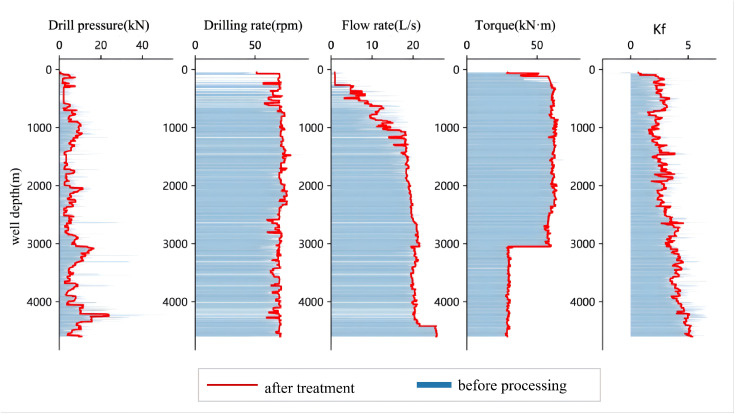
Comparison of Raw Data and Processed Data from Well Zhanghai D.

Based on the original well data and the fused data, a series of comparative plots were generated. As shown in Figs 3, 4, 5, 6, the outlier-processing method applied after data fusion can scientifically and accurately fill in missing values, effectively smooth out “spike” noise points in the raw data, and preserve the overall trends and structural characteristics of the data during this process.

**Fig 7:**
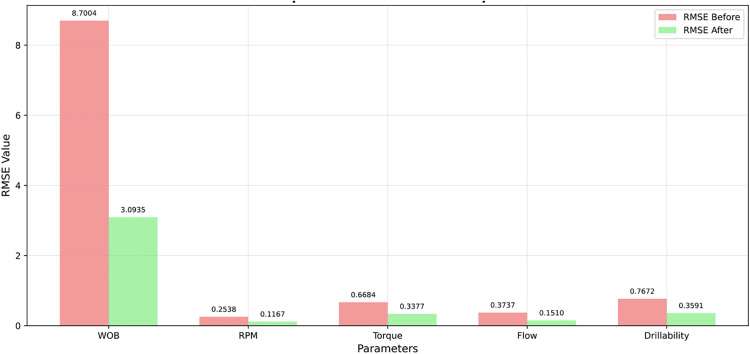
The RMSE comparison diagram of the original and processed data.

Furthermore, to quantitatively evaluate the improvement of key drilling parameters before and after data processing, Fig 7 presents the comparison of RMSE (Root Mean Square Error) values between the original and processed datasets. As shown in the figure, the RMSE values of all parameters decreased significantly after processing: the RMSE of weight on bit (WOB) was reduced from 8. 7004–3. 0935, rotational speed (RPM) from 0. 2538–0. 1167, torque from 0. 6684–0. 3377, flow rate from 0. 3737–0. 1510, and drillability from 0. 7672–0. 3591. The data fusion and smoothing processes effectively reduced noise interference, improved the stability and consistency of each parameter, and provided a more reliable data foundation for subsequent model training.

## 3. Establishment of the hydraulic-mechanical specific energy (HMSE) model

Research on the Mechanical Specific Energy (MSE) model, both domestically and internationally, predominantly centers on aspects like measurement-while-drilling (MWD) techniques, drill bit types, and wear detection. With drilling depths extending into intermediate and deep zones, challenges including high rock hardness, strong abrasiveness, poor drillability, and complex downhole conditions have surfaced, resulting in heightened drilling difficulties and costs. To describe the coupling effect between mechanical and hydraulic energy during the drilling process, this study introduces a hydraulic jet power term into the classical Mechanical Specific Energy (MSE) framework and develops a Hydraulic–Mechanical Specific Energy (HMSE) model. The proposed model comprises three components: (1) classical mechanical specific energy, (2) effective drilling pressure, and (3) the comprehensive energy expression. [31,32].

### 3.1 Classical mechanical specific energy (MSE) model

The classical Mechanical Specific Energy (MSE) serves as a critical metric for quantifying the energy expended by a drill bit per unit volume of rock drilled, thereby acting as a significant indicator of drilling efficiency. This model takes into account the power inputs from both weight on bit and torque, enabling it to accurately reflect the drill bit’s mechanical rock-breaking capability against the formation.


$$MSE=\left(\frac{W_{OB}}{A_{b}}\right)+\frac{120\pi T\cdot RPM}{A_{b}\cdot ROP}$$ (3−1)

In the formula: *MSE* denotes the mechanical specific energy (kPa); *WOB* represents the weight on bit (kN); *T* signifies torque (kN·m); *RPM* indicates the rotational speed of the drill bit (r/min); *ROP* stands for the rate of penetration (m/h); *A*_*b*_ the bottom-hole area (m²) (S1 Appendix).

### 3.2 Hydraulic – mechanical specific energy model

When accounting for the effects of hydraulic jetting, the nozzle jet exerts an impact on the formation while simultaneously generating a reactive force of equal magnitude but opposite direction on the drill bit. This results in the actual effective weight on bit (WOB) acting at the bottom of the well being less than the measured value. To correct for this influence, an energy reduction coefficient, denoted as η, is introduced.


$$W_{OB_{e}}=W_{OB}-\eta F_{j}$$ (3−2)

Here, *η* denotes the energy reduction coefficient (0. 25–0. 40) [33], and *F*_*j*_ represents the nozzle jet impact force (kN). This expression accounts for the influence of the hydraulic jet reaction force acting on the bit, which modifies the effective drilling pressure after considering hydraulic feedback effects.

By taking into account the combined effects of mechanical and hydraulic energy, a hydraulic-mechanical specific energy model can be developed. This model builds upon the classical Mechanical Specific Energy (*MSE*) framework by incorporating an additional term for the jet water power, denoted as *Hp*, which accounts for the supplementary work performed by the hydraulic jet during the rock-breaking process.


$$MSE_{h}=\frac{W_{OB_{e}}+120\pi T\cdot RPM+H_{P}}{A_{b}\cdot ROP}$$ (3−3)

In this expression, *MSE*_*h*_ denotes the hydraulic-mechanical specific energy (kPa); *H*_*p*_ is the effective hydraulic jet power (kW), representing the additional energy input from the hydraulic jet contributing to rock fragmentation.

From a physical perspective, the hydraulic jet creates a localized high-pressure zone at the bottom of the wellbore, weakening the rock’s surface integrity, promoting crack initiation and propagation, and enhancing bottom-hole cleaning to reduce secondary cutting. Overall, this coupled effect slightly decreases the effective drilling pressure while significantly improving rock-breaking efficiency. The proposed model more accurately captures the energy conversion mechanisms under hydraulic–mechanical synergy and provides a theoretical foundation for optimizing drilling parameters.

### 3.3 Determination of key parameters in the model

In the hydraulic-mechanical specific energy model presented in this study, the rate of penetration (ROP) and torque are two critical parameters that remain unknown and must be determined. The ROP is a parameter monitored in real-time during drilling operations. Predicting the ROP for an undrilled section requires relying solely on drilling data collected before the current bit. Without direct measurements of surface or downhole torque from field data, torque must be calculated using two measured values: the bit’s sliding friction coefficient and the weight on bit (WOB). Other parameters, like the jet impact force and bit pressure drop, can be derived from the pre-drilling design parameters that are already known. The drilling parameters that require optimization include the weight on bit (WOB), rotational speed, and flow rate.

#### 3.3.1 Rate of penetration (ROP).

The accuracy of the mechanical drilling rate (ROP) prediction model is crucial for predicting ROP, as it directly impacts the reliability of the parameter optimization model. Accurate ROP values are essential for optimizing drilling parameters. Currently, most ROP prediction studies [34,35] rely on accurate geological and lithological data. However, in certain drilling scenarios, obtaining sufficient drilling and lithological data proves challenging. To tackle this challenge, this section employs both a ternary ROP prediction equation and a BP neural network model to predict drilling rates and compares their outcomes.

(1) Ternary Rate of Penetration (ROP) Prediction Equation

Based on prior research and after a thorough evaluation of the aforementioned factors, a ternary model for the drilling rate equation, incorporating the weight on bit (WOB), rotational speed, and the formation’s drillability index, is proposed [35].


$$V_{op}=C\frac{WOB^{\alpha }RPM^{\lambda }}{K_{f}^{\gamma }}$$ (3–4)

Where: V_op_ represents the predicted mechanical drilling rate (m/h); C is a comprehensive coefficient; WOB denotes the weight on bit (kN); RPM represents the rotational speed (r/min or rpm); K_f_ stands for the drillability index; α is the weight-on-bit exponent; λ is the rotational-speed exponent; γ is the formation-drillability exponent.

The drillability index of a formation, relative to the laboratory-measured rock drillability, is a comprehensive measure. This index effectively predicts drillability during drilling and comprehensively quantifies the bottom-hole formation rock’s resistance to drill bit breakage. The formation’s drillability index is defined as follows: a PDC drill bit takes 128 minutes to drill 1 meter of the formation, corresponding to a drillability coefficient of Level 10. The calculation formula is as follows:


$$K_{f}=log_{2}\left(8*R_{op}\right)$$ (3–5)

Here, R_op_ denotes the drilling time per whole meter as recorded in the logging data, measured in minutes per meter (min/m).

Applying the regression analysis method to the parameters—weight-on-bit exponent, rotational speed exponent, and drillability index—in the comprehensive drilling rate equation enables the derivation of a ternary model for predicting the mechanical drilling rate in this region.

On this basis, the prediction accuracy of the ternary drilling rate equation is assessed by integrating actual drilling data. The subsequent equation is utilized to compute the model’s accuracy:


$$E=1-\frac{\sum_{k=1}^{j}\left(\frac{\left|vop2_{k}-vop1_{k}\right|}{\overset{-}{vop1}}\right)}{m}$$ (3–6)

In the formula, E signifies the model’s computational accuracy, being dimensionless. Variables i and j represent the initial and final well depths, measured in meters (m). VOP1_k_ denotes the original mechanical drilling rate, expressed in meters per hour (m/h). VOP2_k_ stands for the mechanical drilling rate computed by the model, also in meters per hour (m/h). is the average of the original mechanical drilling rates, in meters per hour (m/h). Finally, m indicates the number of data points.

**Fig 8:**
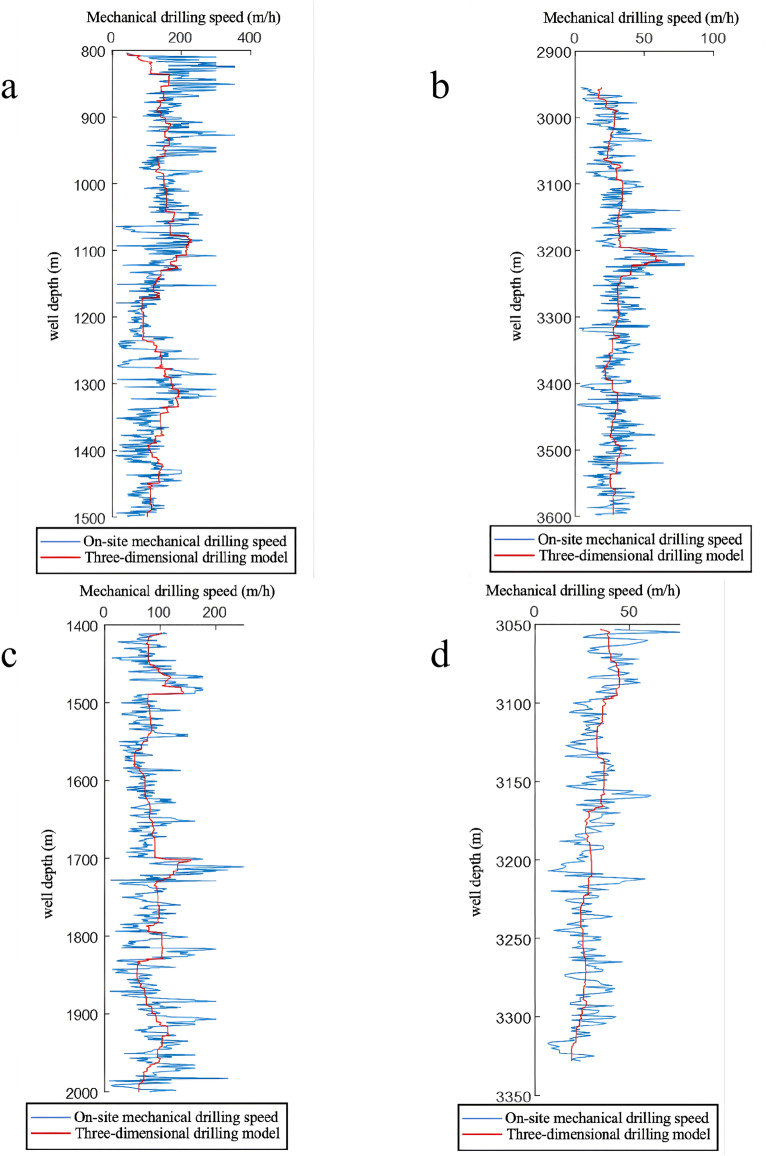
Field Rate of Penetration (ROP) and the Corresponding Prediction Results of the Ternary Model; a) Well Zhanghai A; b)Well Zhanghai C; c)Well Zhanghai C; d)Well Zhanghai D.

As illustrated in Table 11, employing the regression analysis technique for multiple regression on parameters like the weight-on-bit exponent, rotational speed exponent, and drillability index within the comprehensive drilling rate equation enables the derivation of a ternary drilling rate prediction equation, along with the calculation of its accuracy. Upon utilizing the drilling rate prediction equation, the computational accuracy across various well sections is found to be approximately 70%, suggesting a notable discrepancy between the actual and predicted drilling rates (Fig 8).

**Table 11:** Summary of Fitting Parameters for the Ternary Model.

Well number	Beginning depth (m)	Maximum cutting depth (m)	Overall coefficient	Exponent of weight on bit	Exponent of rotary speed	Drillability index	Precision
Well Zhanghai A	804	1500	0.031	0.81	1.09	1.15	0.70
Well Zhanghai B	2955	3598	0.030	0.81	1.09	1.15	0.71
Well Zhanghai C	1410	2000	0.057	0.81	1.09	1.15	0.71
Well Zhanghai D	3053	3328	0.190	0.81	1.09	1.15	0.76

(2) BP Neural Network Model

The use of machine learning algorithms for ROP prediction has become an important data-driven approach to improving drilling efficiency. Early studies, such as those by Bilgesu et al. [36], explored the potential application of neural networks in ROP prediction. In recent years, with the advancement of algorithms, researchers such as Al-AbdulJabbar et al. [37] have further demonstrated the superior performance of artificial intelligence models in achieving high-accuracy ROP predictions. These developments provide a solid theoretical and practical foundation for the application of the BP neural network model in this study.

The BP (Back Propagation) neural network algorithm comprises two primary components: the forward propagation of signals and the backward propagation of errors. During forward propagation, signals are transmitted from the input layer, through one or more hidden layers, to the output layer, resulting in the network’s actual output. If the actual output deviates from the desired output, backward propagation of errors is initiated. During the backward propagation phase, the output error is propagated backwards from the output layer, through the hidden layer(s), to the input layer, generating error signals for each unit in every layer. These error signals are subsequently utilized to adjust the network’s connection weights. This iterative process is repeated, with the connection weights being continually adjusted until the error falls below a predefined threshold or until a preset number of learning iterations is completed [38,39].

Utilizing historical data from other wells and pre-drilling information for the current well, the front-section historical data and pre-bit data serve as the training set, whereas the back-section historical data and data for the upcoming well (to be drilled) constitute the test set. In accordance with the mapping relationship outlined in the Rate of Penetration (ROP) equation, mechanical specific drilling rate (ROP), weight-on-bit (WOB), rotational speed, and the extremum of formation drillability are chosen as input parameters, with the mechanical specific drilling rate (ROP) serving as the output, to develop a BP neural network prediction model (Table 12).

**Table 12:** Training Data for Well Zhanghai A (Partial).

Well depth(m) (m)	Drill pressure (kN)	Revolution speed (rpm)	Drillability	Mechanical drilling speed (m/h)
2700	80.34	81.54	3.56	38.22
2701	80.34	81.54	3.56	41.67
2702	84.63	81.54	3.53	31.41
2703	85.33	81.54	3.53	23.26
2704	90.55	81.48	3.53	17.24
2705	90.55	81.48	3.53	22.39
2706	93.95	81.5	3.53	19.11
2707	96.51	81.48	3.53	68.97
2708	96.51	81.54	3.53	66.67
2709	99.26	81.48	3.53	71.43
2710	98.56	81.54	3.53	61.86
2711	98	81.54	3.53	43.80
2712	98	81.5	3.53	41.67
2713	98	81.54	3.56	40.82
2714	98	81.54	3.45	34.48
2715	98.07	81.54	3.23	51.28
2716	98.07	81.54	3.23	57.69
2717	98.07	81.7	3.06	68.97
2718	98.07	81.7	2.96	61.86
2719	98.07	81.7	3.06	71.43
2720	98.07	81.7	3.06	66.67

**Fig 9:**
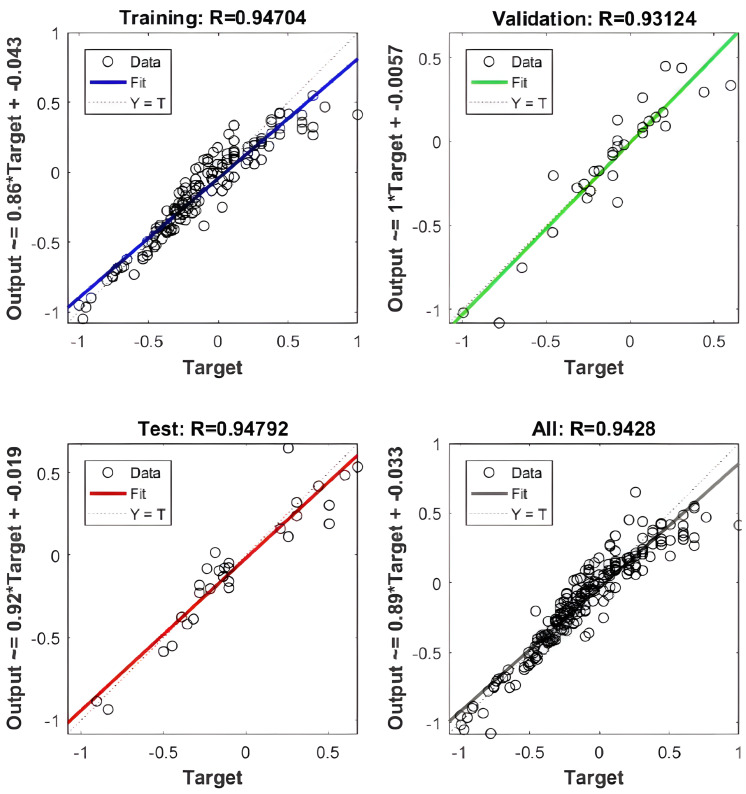
Regression Analysis of the Sample Set in the BP Neural Network.

The Trainglm function in MATLAB is employed to implement a dynamically adaptive learning rate algorithm. After multiple rounds of training, it was observed that the BP neural network achieved optimal convergence at the ninth iteration. As shown in the sample set regression analysis (Fig 9), the samples exhibit a strong correlation, with a high correlation coefficient and an R value exceeding 0. 9. This indicates that the neural network model trained on the data performs well in predicting the rate of penetration (ROP).

**Fig 10:**
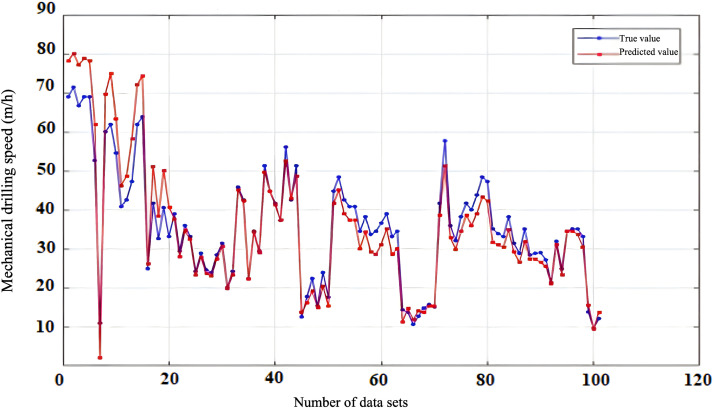
Prediction Performance of Rate of Penetration (ROP) for Well Zhanghai A.

**Fig 11:**
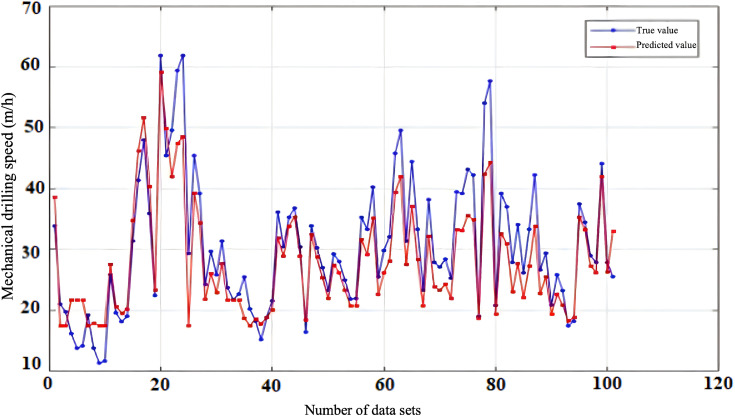
Prediction Performance of Rate of Penetration (ROP) for Well Zhanghai B.

**Fig 12:**
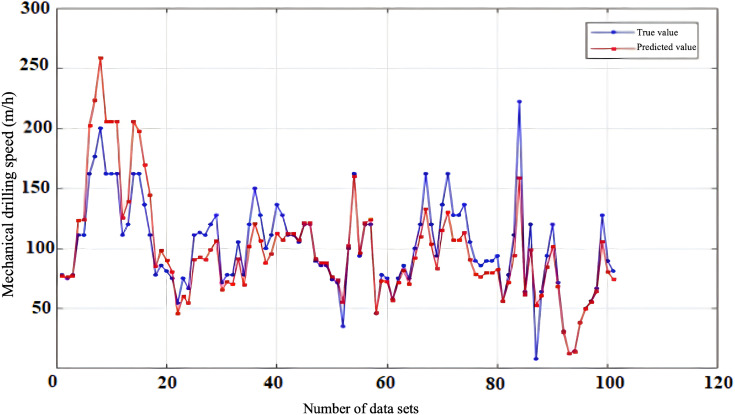
Prediction Performance of Rate of Penetration (ROP) for Well Zhanghai C.

To evaluate the reliability and accuracy of the BP neural network model, 300 sets of historical drilling data were collected from various intervals of four wells in the Zhanghai oilfield. The initial 200 data sets were employed for model training, and the subsequent 100 sets were used for validating the predictive performance. Fig 10, 11, 12, 13 depict the stress-strain curves comparing the actual values with those predicted by the BP neural network, clearly demonstrating a good agreement between the predicted and actual results. This finding suggests that, following training, the BP neural network model designed for deep-well applications can accurately forecast the rate of penetration (ROP) and exhibits robust generalization capabilities.

**Fig 13:**
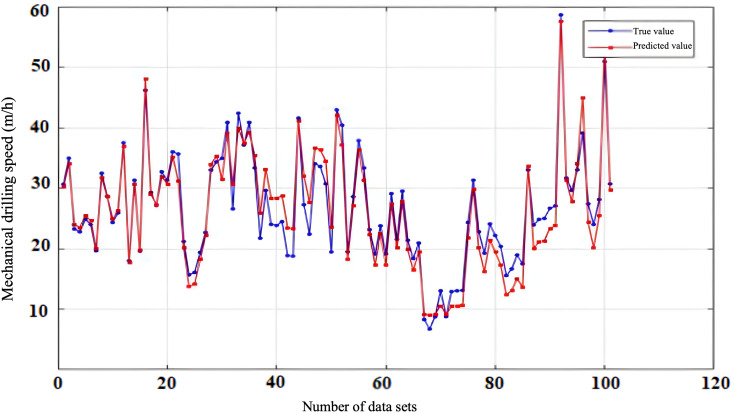
Prediction Performance of Rate of Penetration (ROP) for Well Zhanghai D.

Table 13 presents the confidence intervals for the Root Mean Square Error (RMSE), Mean Absolute Percentage Error (MAPE), along with the determination coefficient (R^2^) for the prediction outcomes generated by the BP neural network model. Analyzing the data in the table, it becomes evident that the BP neural network model’s predictions of drilling speed exhibit minimal fluctuations within the RMSE and MAPE confidence intervals. This observation underscores the model’s stable prediction error, robust performance, and overall efficacy within an optimal range, thereby enabling more precise forecasting of drilling speed values [39].

**Table 13:** Evaluation Indicators for the BP Neural Network Model.

Well section	RMSE Confidence Interval	MAPE (%) Confidence Interval	R²
Well A 2700–3000 m	8. 72 ~ 10. 45	8. 61% ~ 10. 32%	0. 942
Well B 3300–3500 m	8. 90 ~ 10. 67	8. 83% ~ 10. 92%	0. 913
Well C 1700–2000 m	8. 84 ~ 10. 34	8. 72% ~ 10. 87%	0. 925
Well D 3050–3350 m	7. 85 ~ 9. 30	7. 93% ~ 9. 41%	0. 956

(3) Comparison of Prediction Accuracy between the Two Models

The ternary drilling rate prediction model and the BP neural network model were employed to make predictions using data from Wells Zhanghai A, B, C, and D. Subsequently, the predicted values were compared with the actual data, and the accuracy of the predictions was calculated to assess and contrast the performance of the models, as detailed in the Table 14 below.

**Table 14:** Comparison of Prediction Accuracy.

well section	Three-dimensional drilling rate equation prediction model	Bp neural network prediction model
Well Zhanghai A, depth range of 2700–3000 meters	0. 70	0. 94
Well Zhanghai B, depth interval of 3300–3500 meters	0. 71	0. 91
Well Zhanghai C, depth range of 1700–2000 meters	0. 71	0. 92
Well Zhanghai D, depth range of 3050–3350 meters	0. 76	0. 95

As shown in the Table 14, the prediction accuracy of the BP neural network model across the four well sections is significantly higher than that of the three-dimensional drilling rate equation model, indicating superior predictive performance. Furthermore, to determine whether the improvement achieved by the BP model is statistically significant, paired-sample t-tests were conducted on the RMSE and MAPE values of the two models. The results are as follows: the mean improvement in RMSE was 1. 84 m/h (t = 3. 72, p = 0. 021); the mean improvement in MAPE was 1. 18% (t = 4. 05, p = 0. 016); and the mean increase in the coefficient of determination (R²) was 0. 027 (t = 3. 94, p = 0. 018). Since all p-values are below 0. 05, it can be concluded that the improvement of the BP model over the multiple linear regression (MLR) model is statistically significant. This demonstrates that the BP neural network has clear advantages in capturing the nonlinear relationships among drilling parameters. Therefore, this study adopts the BP neural network model as the primary method for predicting the rate of penetration (ROP) in unknown well sections.

#### 3.3.2 Torque.

In calculations, torque (T) serves as a primary variable, commonly acquired through comprehensive logging tools or Measurement While Drilling (MWD) systems [40,41]. However, the data recorded at drilling sites primarily consist of weight on bit (WOB), rotational speed, rate of penetration (ROP), and bit diameter. Direct measurements of surface or downhole torque are frequently unavailable. Under these circumstances, torque must be computed based on the available measured data. Specifically, bit torque can be determined using the bit’s sliding friction coefficient and the weight on bit.

In accordance with the pertinent theorems of double integrals, the torque (T) encountered during the drilling process may be formulated as [42]:


$$T=\int {\mathrm{\backslash nolimits}}_{0}^{\frac{d_{b}}{\text{2}}}\int {\mathrm{\backslash nolimits}}_{0}^{2\pi }\rho^{2}\frac{4\mu WOB}{\pi d_{B}^{2}}d\rho d\theta =\int {\mathrm{\backslash nolimits}}_{0}^{\frac{d_{b}}{\text{2}}}\frac{8\mu WOB}{d_{B}^{2}}\rho^{2}d\rho =\frac{8\mu WOB}{d_{B}^{2}}{\left(\frac{\rho^{3}}{\text{3}}\right)}_{0}^{\frac{d_{B}}{\text{2}}}=\frac{\mu WOBd_{B}}{36}$$ (3–7)

In the formula: dB denotes the bit radius (in meters, m); μ represents the sliding friction coefficient of the bit; WOB signifies the weight on bit (in kilonewtons, kN); and ρ indicates the distance from a specific point to the center (in meters, m).

Upon substituting Equation into the original model, the following is derived:


$$MSE_{h}=\frac{4\left(WOB-\eta F_{j}\right)}{\pi d_{B}^{2}}+\frac{40\mu RPMWOB}{3d_{B}ROP}+\frac{14400\eta \Delta P_{b}Q}{\pi d_{B}^{2}ROP}$$ (3–8)

MSE_h_ denotes the hydraulic-mechanical specific energy (kPa); WOB signifies the measured weight applied to the bit (kN); η represents the energy reduction coefficient, a dimensionless parameter typically ranging between 25% and 40%, The value range of the energy reduction coefficient η in the model is set between 25% and 40%. This range is determined based on classical drilling engineering theory. As noted by Bourgoyne et al. [43], due to the energy dissipation of the fluid jet within the wellbore, typically only a portion of the nozzle’s hydraulic power can effectively contribute to bottom-hole cleaning and assist in rock-breaking operations; Q denotes the drilling fluid flow rate via the bit nozzles (L/s); RPM indicates the bit’s rotational speed, in revolutions per minute (rpm); ROP signifies the penetration rate (m/h); dB denotes the bit diameter (m); μ represents the sliding friction coefficient for the bit; ΔP_b_ denotes the pressure drop across the bit (MPa); F_j_ represents the jet impact force at the nozzle exit (kN).

#### 3.3.3 Mechanical specific energy baseline.

The mechanical specific energy (MSE) baseline defines the upper limit of rock-breaking efficiency during drilling optimization. It serves as a benchmark for analyzing the specific energy curve. Comparing the MSE baseline with the actual specific energy curve during drilling allows for the assessment of drilling parameter optimization effectiveness. A larger deviation between the actual specific energy curve and the MSE baseline correlates with lower rock-breaking efficiency, suggesting the necessity of adjusting drilling parameters. Well logging data effectively capture the physical and mechanical characteristics of formation rocks. These data enable the characterization of the mechanical parameters of the rocks. Well logging data necessary for compressive strength measurement encompass acoustic travel time, shale content, and density.

The conversion formula for longitudinal waves is presented below:


$$V_{p}=0.3084/AC\times 1000$$ (3-9)

$$\Delta t_{p}=1/V_{p}$$ (3-10)

In the formula, AC denotes acoustic slowness (μs/m), V_P_ represents the P-wave velocity (km/s), and signifies P-wave slowness (ms/m).

The conversion formula for shear waves is presented as follows:


$$V_{s}=0.704V_{p}-0.554$$ (3-11)

$$\Delta t_{s}=1/V_{s}$$ (3-12)

In the formula, V_s_ corresponds to the shear-wave velocity (km/s), and signifies the shear-wave slowness (ms/m).

young’s modulus:


$$E=\left(\frac{\rho }{\Delta t_{s}^{2}}\right)\left[\frac{3\Delta t_{s}^{2}-4\Delta t_{p}^{2}}{\Delta t_{s}^{2}-\Delta {t_{p}}^{2}}\right]$$ (3-13)

The compressive strength of rocks is determined via an empirical formula [44]. For instance, the compressive strength of sandstone can be calculated as follows:


$$CCS=0.035V_{p}-31.5$$ (3-14)

The compressive strength of mudstone is as follows:


$$CCS=7.97E^{0.91}$$ (3-15)

In the formula, E corresponds to Young’s modulus of elasticity (GPa), and ρ indicates the density (g/cm³).

Intervals from Well Zhanghai A and Well Zhanghai B were selected to compute the mechanical specific energy baseline. The lithological features of these intervals are detailed in Table 15 (for Well Zhanghai A) and Table 16 (for Well Zhanghai B).

**Table 15:** Lithology Table for Part of the Strata in Well Zhanghai A.

Stratification	Deep bottom boundary(m)	Lamination thickness(m)	Lithological characteristics
Member 1 of the Shahejie Formation	3442	334	Grayish-brown fluorescent marl and grayish-brown fluorescent calcareous mudstone.
Member 2 of the Shahejie Formation	3776	334	Light gray fluorescent fine-grained sandstone.
Member 3 of the Shahejie Formation	3974	198	Light gray fluorescent calcareous sandstone.

**Table 16:** Lithology Table for Part of the Strata in Well Zhanghai B.

Stratification	Deep bottom boundary(m)	Lamination thickness(m)	Lithological characteristics
Pingyuan Formation	339	339	A suite of brownish-yellow silty clay, interbedded with various sand layers of different grain sizes. It contains fossils such as sporopollen, gastropods, and bivalves.
Minghuazhen Formation	2106	1767	Earthy yellow and reddish-brown mudstone and sandy mudstone. The upper section exhibits coarser grain size and lighter color, containing iron-manganese nodules and calcareous concretions, while the lower section displays finer grain size and darker color.
Guantao Formation	2538	432	Grayish-white, grayish-green, dark maroon sandstone, and mudstone.

**Fig 14:**
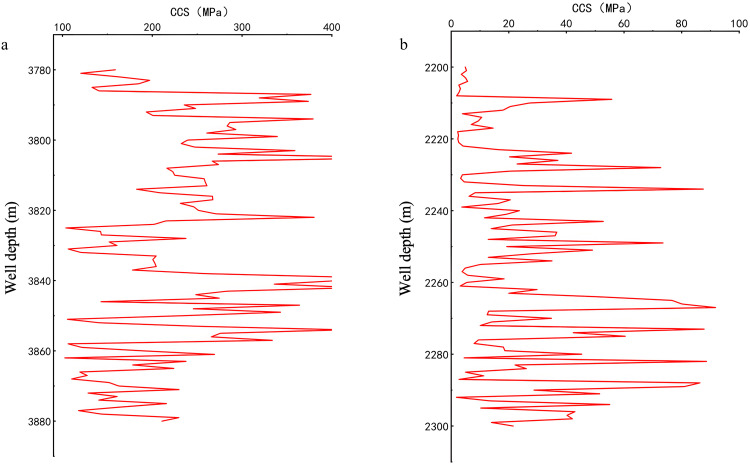
Mechanical Specific Energy Baseline; a) Well Zhanghai A; b) Well Zhanghai B.

A method utilizing well logging data is employed to calculate the compressive strength of rocks. Based on the lithological characteristics of various intervals in Well Zhanghai A and Well Zhanghai B, the appropriate compressive strength formulas are used to compute CCS. In the 3780m-3880m interval of Well Zhanghai A, which predominantly consists of sandstone. In the 2200m-2300m interval of Well Zhanghai B, predominantly composed of mudstone. The mechanical specific energy baseline is depicted in Fig 14.

## 4. Influencing factors of the hydraulic-mechanical specific energy model

During drilling, multiple factors affect the variability of the Mechanical Specific Energy (MSE). The MSE model indicates that MSE is directly linked to adjustable drilling parameters, including weight on bit (WOB), rotational speed, and flow rate. Further analysis is required to understand the impact of these parameters on MSE. Additionally, there are unaccounted-for factors, like bit balling and bit dulling, that can also influence MSE variability. We must also analyze these factors and implement appropriate countermeasures.

### 4.1 Correlation analysis of drilling parameters

**Fig 15:**
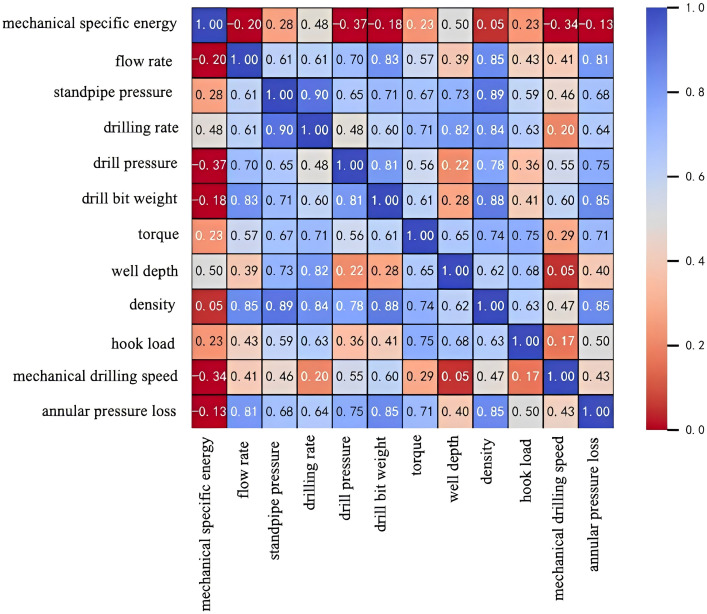
Correlation Analysis of Parameters in Drilling Data.

Feature parameters, including well depth, weight on bit (WOB), rotational speed, flow rate, rate of penetration (ROP), standpipe pressure, bit pressure drop, torque, drilling fluid density, hook load, and annular pressure loss, were chosen for analysis. The correlations between these feature parameters and Mechanical Specific Energy (MSE) were computed for approximately 4,000 sets of historical well data collected from Well Zhanghai A, as detailed in Table 17. Subsequently, a heatmap was produced using Python’s Seaborn library, as illustrated in Fig 15.

**Table 17:** Data Table for Correlation Analysis (Partial).

Well depth(m)	2600	2601	2602	2603	2604	2605	2606	2607	2608	2609	2700
Mechanical specific energy (MPa)	397	279	301	365	300	334	429	515	407	484	652
Flow rate (L/s)	29. 87	29. 87	29. 87	29. 87	29. 87	29. 87	29. 87	29. 87	29. 87	29. 87	29. 87
Standpipe pressure (MPa)	14	12	14	13	13	12	12	12	13	12	12
Revolution speed (rpm)	81. 45	81. 46	81. 47	81. 48	81. 49	81. 50	81. 51	81. 53	81. 54	81. 55	81. 56
Drill pressure(kN)	102. 88	102. 88	103. 23	103. 23	102. 88	102. 88	102. 88	102. 88	102. 88	102. 88	102. 88
Bit nozzle pressure-drop (MPa)	3. 35	3. 35	3. 35	3. 35	3. 35	3. 35	3. 35	3. 35	3. 35	3. 35	3. 35
Torque (kN·m)	21. 45	23. 52	23. 07	21. 96	21. 85	22. 4	22. 9	22. 23	22. 69	22. 14	21. 33
Density (g/cm^3^)	1. 07	1. 08	1. 08	1. 07	1. 08	1. 08	1. 08	1. 08	1. 08	1. 07	1. 08
Hook load (kN)	919	926	881	902	903	902	896	900	901	904	910
Mechanical drilling speed (m/h)	48. 39	68. 97	63. 83	52. 63	63. 83	57. 69	44. 78	37. 27	47. 24	40. 00	29. 41
Annular pressure loss (MPa)	0. 85	0. 85	0. 85	0. 85	0. 85	0. 85	0. 85	0. 85	0. 85	0. 85	0. 85

As depicted in Fig 15, the parameters strongly positively correlated with Mechanical Specific Energy (MSE) are well depth, rotational speed, standpipe pressure, hook load, and torque, listed in descending order of correlation strength. In contrast, the parameters exhibiting a strong negative correlation with MSE are weight on bit (WOB), rate of penetration (ROP), flow rate, bit pressure drop, and annular pressure loss. Mutual information analysis between each parameter and MSE reveals that the primary factors influencing MSE are weight on bit (WOB), rotational speed, and flow rate. Further investigation will explore the specific impact mechanisms of WOB, rotational speed, and flow rate on MSE.

### 4.2 Drilling pressure

During drilling, the PDC bit penetrates the formation under the influence of the weight on bit (WOB). The penetration depth of the bit into the formation is strongly correlated with the WOB magnitude. Consequently, the WOB is identified as a primary factor influencing the Mechanical Specific Energy (MSE). Since field-measured WOB values vary continuously, the WOB data are segmented for analysis. Table 18 presents a summary of the data analyzing the sensitivity of MSE to WOB. While maintaining constant rotational speed and flow rate, the WOB values selected for analysis are 50 kN, 100 kN, 200 kN, and 300 kN.

**Table 18:** Sensitivity Analysis of Weight on Bit (WOB) to Mechanical Specific Energy and Field Drilling Data.

Category	Drill pressure(kN)	Revolution speed(rpm)	Flow rate(L/s)	Torque(kN·m)	Bit diameter(mm)	Drilling mud density(g/cm^3^)	Total area of nozzle (mm^2^)	Drill bit category
Sensitivity analysis	50	60	65	7	311. 1	1. 1	1407. 43	PDC
	100							
	200							
	300							
Field data	150-200	82	65	10 ~ 15	311. 1	1. 1	1407. 43	PDC
	200-250							
	250-300							

**Fig 16:**
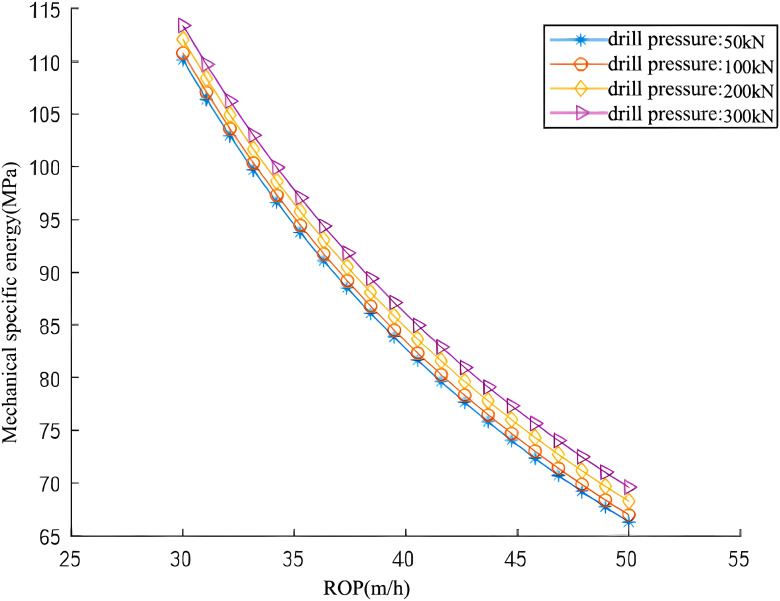
Variation Curve of Mechanical Specific Energy with Rate of Penetration (ROP) under Different Weight on Bit (WOB) Conditions.

**Fig 17:**
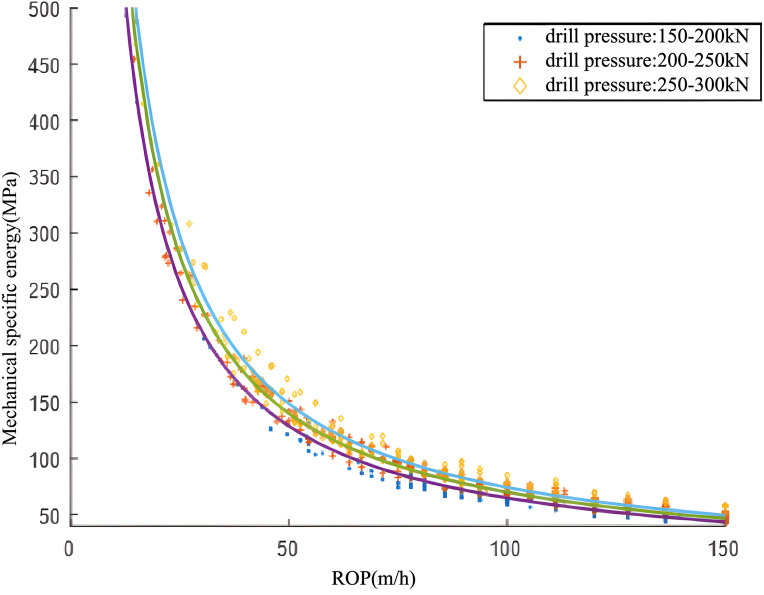
Variation Curve of Mechanical Specific Energy with Rate of Penetration (ROP) under Different Weight on Bit (WOB) Conditions.

As illustrated in Figs 16 and 17, analysis of actual drilling data reveals that the Mechanical Specific Energy (MSE) decreases as the Rate of Penetration (ROP) increases. A comparison of MSE and ROP curves, derived from tests using the same PDC bit under varying WOB conditions, indicates that MSE increases with WOB at a constant ROP. Furthermore, these curves delineate two distinct drilling regimes. Fig 16 showcases an inefficient drilling regime with low ROP and high MSE, in contrast to Fig 17, which illustrates an efficient drilling regime with high ROP and low MSE. The key factors contributing to the efficient drilling regime are outlined below:Initially, under favorable bottomhole cleaning conditions, an increase in WOB facilitates deeper PDC bit penetration into the formation, consequently boosting the ROP. Secondly, with rising WOB, the bottomhole rock lithology shifts from brittle to pseudo-plastic. The PDC bit’s unique rock-breaking mechanism enables efficient cutting under these conditions. However, WOB cannot be increased without limit and is constrained by factors including bit strength, formation characteristics, wellbore integrity, and the effectiveness of bottomhole rock cleaning.

### 4.3 Rotating speed

Both excessively high and low rotational speeds have a substantial impact on the Mechanical Specific Energy (MSE). When the rotational speed is excessively high, the bit’s penetration depth into the formation is limited, and the weight on bit (WOB) cannot be substantially increased without risking drill pipe failure or bit bouncing. Conversely, at excessively low rotational speeds, the drilling tool’s penetration rate diminishes, thereby reducing work efficiency. Table 19 summarizes data analyzing the sensitivity of MSE to variations in rotational speed. Maintaining constant WOB and flow rate, the analysis focuses on rotational speeds of 20 rpm, 40 rpm, 60 rpm, and 80 rpm.

**Table 19:** Sensitivity Analysis of Rotational Speed on Mechanical Specific Energy.

Drill pressure (kN)	Revolution speed (rpm)	Flow rate (L/s)	Torque (kN·m)	Bit diameter (mm)	Drilling mud density (g/cm3)	Total area of nozzle (mm2)	Drill bit category
200	20	65	7	311. 1	1. 1	1047. 43	PDC
	40						
	60						
	80						

**Fig 18:**
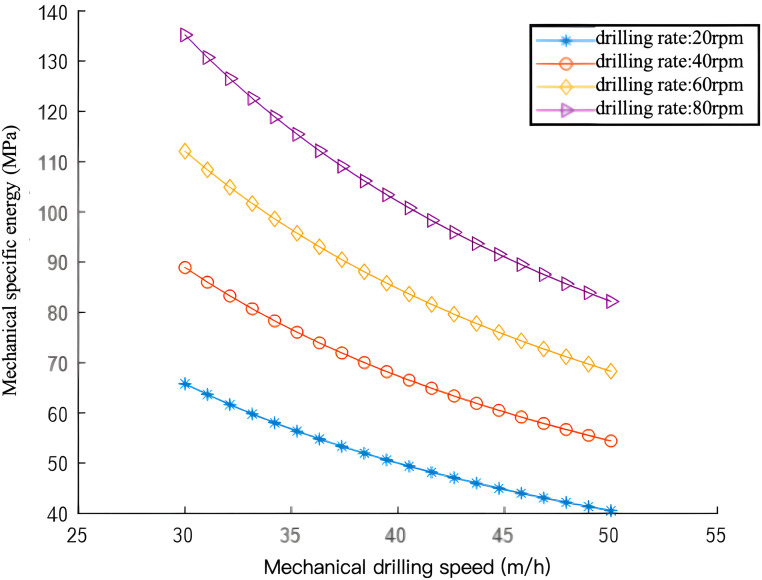
Variation Curve of Mechanical Specific Energy with Rate of Penetration (ROP) at Different Rotational Speeds.

Fig 18 depicts the results. At a constant rate of penetration (ROP), MSE rises with an increase in rotational speed. In field drilling, the maximum rotational speed of the drilling rig’s rotary table is constrained primarily by the following factors:(1) Bit type: PDC bits vary in cutting tooth size and strength, each with a specified maximum rotational speed. (2) Drill string strength: Excessively high rotational speeds can cause drill string torsional failure due to the additional torque from bit sticking, thereby limiting the maximum rotational speed. (3) Formation lithology: In soft formations, ROP increases proportionally with rotational speed. However, in harder formations, this proportionality no longer holds. This occurs because at higher rotational speeds, the bit impacts the rock more frequently per unit time, reducing drill tooth-formation contact time. If contact time drops below the time needed for rock fragmentation, drill teeth penetration effectiveness diminishes, significantly reducing rock-breaking efficiency.

### 4.4 Flow

Drilling fluid, used in drilling operations, serves several key functions:(1) It cleans the well bottom, suspends, and transports cuttings to keep the borehole clear; (2) It balances formation pressure, stabilizes the wellbore wall, and prevents well collapse, blowouts, and lost circulation; (3) It transmits hydraulic power to aid the bit in rock fragmentation; (4) It cools the bit and drilling tools to prevent accelerated wear of cutting teeth caused by frictional heat, necessitating efficient hydraulic performance for rapid bit cooling; (5) It is used for geological and gas logging. Drilling fluid significantly impacts drilling operations and is a key determinant of drilling efficiency. Table 20 summarizes data on the sensitivity of Mechanical Specific Energy (MSE) to drilling fluid flow rate. Under constant weight on bit (WOB) and rotational speed, the flow rates tested were 10 L/s, 30 L/s, 50 L/s, and 70 L/s.

**Table 20:** Sensitivity Analysis of Flow Rate on Mechanical Specific Energy.

Drill pressure (kN)	Revolution speed (rpm)	Flow rate (L/s)	Torque (kN·m)	Bit diameter (mm)	Drilling mud density (g/cm^3^)	Total area of nozzle (mm^2^)	Drill bit category
200	60	10	7	311. 1	1. 1	1047. 43	PDC
		30					
		50					
		70					

**Fig 19:**
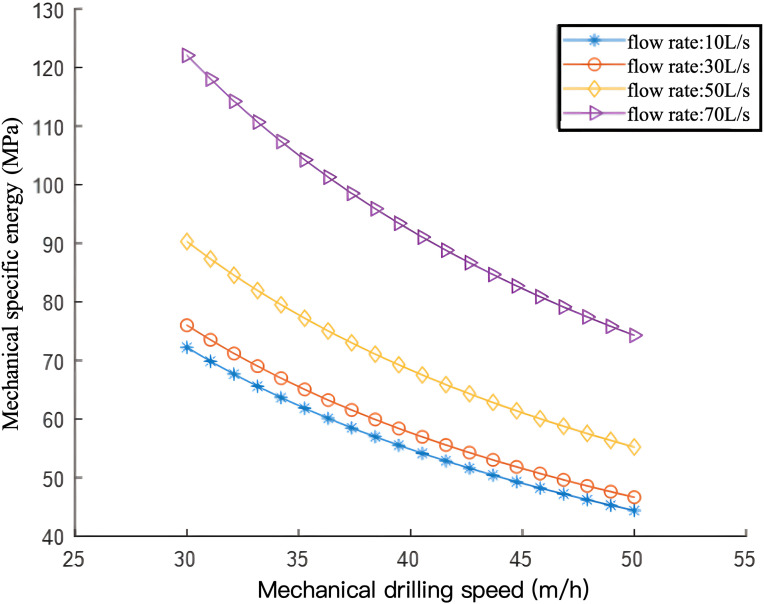
Curves Depicting the Relationship Between Mechanical Specific Energy and Rate of Penetration (ROP) at Different Flow Rates.

Results are presented in Fig 19. Under the same mechanical drilling rate, an increase in flow rate leads to a rise in Mechanical Specific Energy (MSE). Flow rate magnitude is constrained by the following factors:(1) Drilling fluid density: If the drilling fluid density is too low, the flow rate should be increased to maintain formation pressure balance, stabilize the wellbore, and ensure effective borehole cleaning. Otherwise, inefficient rock fragmentation, reduced bit efficiency, and an increased risk of blowouts, well collapse, and stuck pipe incidents may occur. Conversely, if the drilling fluid density is too high, the flow rate should be decreased to prevent excessive formation leakage and damage to hydrocarbon-bearing zones. (2) Pipeline pressure capacity: A higher flow rate increases pipeline pressure. Exceeding the pipeline’s maximum pressure capacity may result in rupture and drilling accidents. (3) Mud pump power: The mud pump directly controls the flow rate through a gearbox or hydraulic motor that adjusts its speed. Additionally, the mud pump is equipped with a flowmeter and pressure gauge for on-site personnel to monitor and control pump pressure and flow rate changes accurately.

### 4.5 Drill bit mud bag

**Fig 20:**
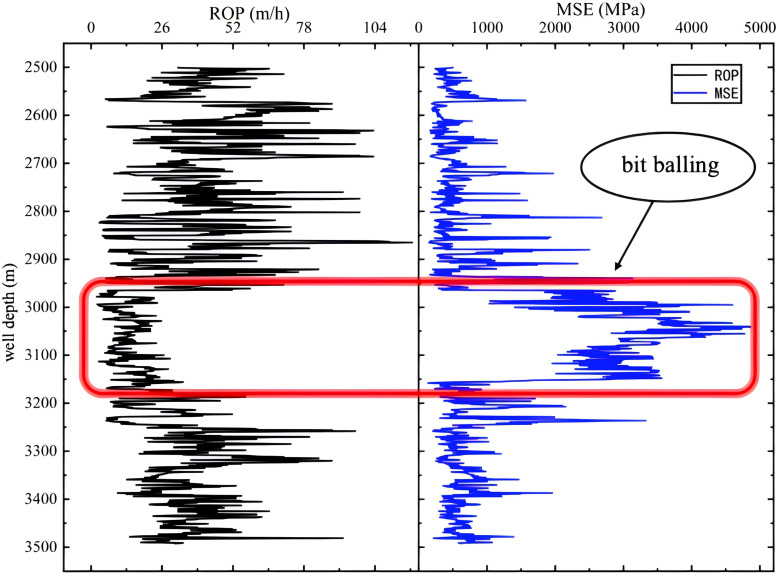
Corresponding Curve of Rate of Penetration (ROP) versus Mechanical Specific Energy (MSE) in the Case of Bit Balling.

Bit balling is the phenomenon where the drill bit surface accumulates a mud coating, which can happen during drilling and tripping operations. The primary causes of bit balling encompass geological conditions, mud characteristics, engineering methodologies, bit type selection, and operational skill [45]. Common signs of PDC bit balling include: (1) a notable reduction in drilling speed coupled with an extended drilling duration; (2) minimal change in the rate of penetration (ROP) despite adjustments to the weight on bit (WOB); (3) negligible change in ROP even when formation properties vary (Fig 20).

Upon encountering bit balling, the initial action is to promptly halt drilling and introduce a cleaning agent into the wellbore as soon as possible to clean the drill bit. Additionally, the flow rate should be elevated to boost the efficiency of hydraulic flushing. The drill bit should be raised to disengage from the well bottom. Subsequently, the rotational speed should be increased to generate a stronger centrifugal force, aiding in the expulsion of mud clumps. Extensive vertical movement should be performed. Following this, the drill bit should be returned to the well bottom without rotating the turntable for 5–10 minutes of circulation. These operations should be repeated. If these operations fail to yield results within two circulation cycles, it is recommended to consider retrieving the drill string.

## 5. Methods for drilling parameter optimization and case study analysis

Scientific design of drilling parameters optimizes the potential energy of these parameters, thereby enhancing the energy efficiency of drilling tools. The hydraulic-mechanical specific energy model precisely quantifies the mechanical energy required to fracture a unit volume of rock. A lower specific energy value signifies improved rock-breaking efficiency by the drill bit, optimized drilling parameters, and enhanced overall drilling efficiency. This chapter employs the previously established hydraulic-mechanical specific energy model as the objective function, optimizing parameters such as weight on bit (WOB), rotational speed, and flow rate. The simulated annealing algorithm [46] will be applied to determine the optimal drilling parameters that minimize mechanical specific energy within the equipment’s operational constraints. A case study will then be conducted to validate and analyze the results.

### 5.1 Parameter preparation and constraint conditions

The data used in this chapter consist of historical records from Well Zhanghai A. Some data have been processed using the drilling data fusion technique previously described. The optimization objective is the mechanical specific energy (MSE) model, as defined. Selected basic parameter values for the model are listed in Table 21. This section focuses on optimizing the 2700m-2800m interval of Well Zhanghai A. The basic parameters remain constant within this interval. The Rate of Penetration (ROP) is predicted using a BP neural network model, and the specific values are detailed in Table 22.

**Table 21:** Partial Parameter Values for the Mechanical Specific Energy Model.

Energy reduction coefficient	0. 3
Drilling mud density(g/cm^3^)	1. 25
Total cross-sectional area of nozzle outlet(mm^2^)	896. 14
Bit diameter(m)	0. 2159
Sliding friction coefficient of drill bit	0. 4

**Table 22:** Predicted Values of Rate of Penetration (ROP) for Well Zhanghai A (Partial).

Well depth(m)	The predicted value of mechanical drilling speed(m/h)
2800	68. 96
2801	71. 42
2802	66. 66
2803	68. 96
2804	68. 96
2805	52. 63
2806	10. 94
2807	60. 00
2808	61. 85
2809	54. 54
2810	40. 81

**Fig 21:**
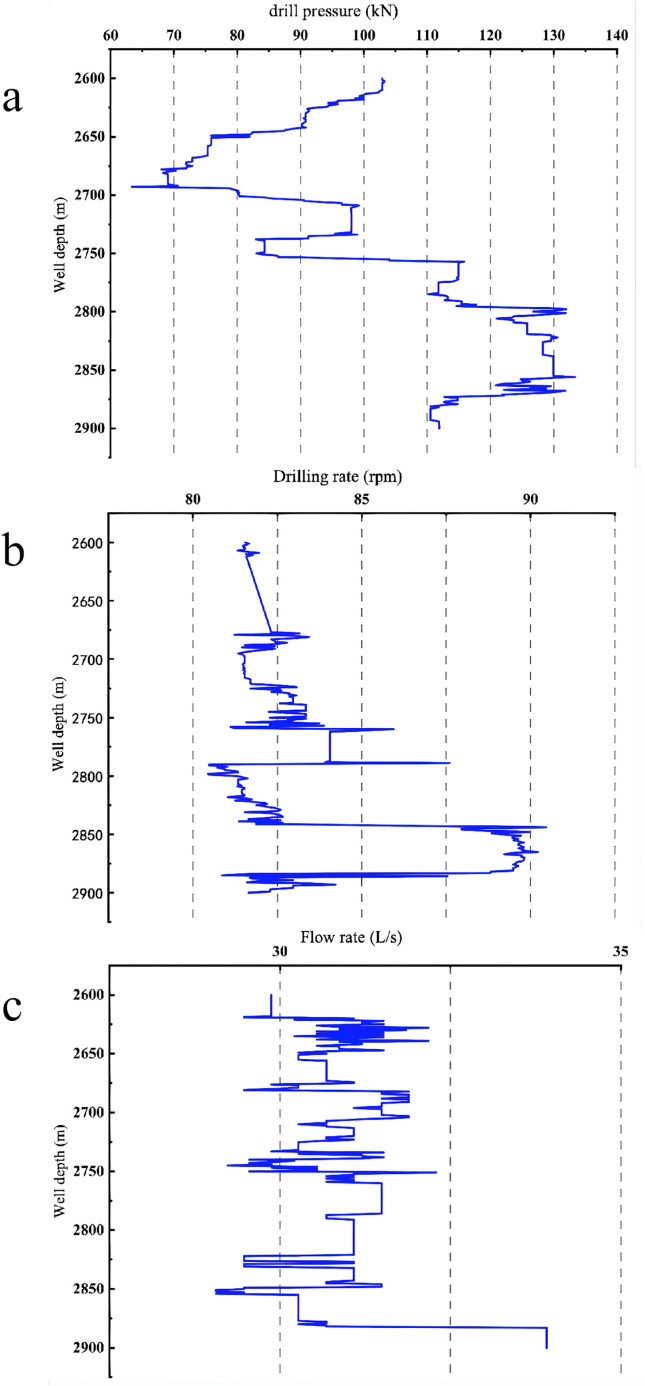
Limit Values of Parameters for Well Zhanghai A.

The weight on bit (WOB), rotational speed, and flow rate are designated as the drilling parameters for optimization. During optimization, it is crucial to determine the constraint ranges for these parameters. Exceeding these optimization ranges may result in breaching the equipment’s operational limits during drilling, presenting engineering hazards. During drilling of Well Zhanghai A, the upper and lower limits for WOB, rotational speed, and flow rate within the 2700m-2800m interval are depicted in the Fig 21, approximating the operational limits of the on-site drilling equipment.

The maximum and minimum values that can be achieved by optimizing the drilling parameters are listed in Table 23.

**Table 23:** Parameter Constraint Conditions.

	Drill pressure (kN)	Revolution speed(rpm)	Flow rate (L/s)
Minimum value	60	70	20
Maximum value	140	100	40

### 5.2 Optimization methods for drilling parameters

Parameter optimization involves finding the optimal parameter combination to achieve the desired target effect. Most machine learning problems can be viewed as parameter optimization tasks aimed at minimizing the loss function. The variation in mechanical specific energy (MSE) is complex, with local optima present in high-dimensional spaces. The MSE-based optimization objective aims to identify the globally optimal drilling parameter combination.The use of intelligent algorithms for dynamic optimization of drilling parameters to enhance energy efficiency has become a prominent research focus. For instance, Kunshin et al. [4] recently developed an MSE-based drilling energy efficiency monitoring and prediction technology, demonstrating the significant application potential of intelligent algorithms in this field. Considering the continuous variability of drilling parameter optimization, this paper employs the simulated annealing algorithm to solve the parameter combination problem, as it offers greater advantages in tackling complex nonlinear optimization challenges.

Firstly, the objective function is set as the hydraulic-mechanical specific energy model introduced earlier. The parameters to be optimized include the weight on bit (WOB), rotational speed, and flow rate. The aim is to identify the drilling parameter combination that yields the minimum mechanical specific energy. Consequently, the mathematical formulation of this optimization problem is presented as:


$$min\{MSE=f(WOB,RPM,Q)\;s.t.\;\{\;\;\;\begin{array}{c} {}*20clb_{WOB}\leq WOB\leq ub_{WOB}\\ lb_{RPM}\leq N\leq ub_{RPM}\\ lb_{Q}\leq Q\leq ub_{Q} \end{array}$$ (5−1)

Moving forward, all translation requests I make do not require markdown formatting. I will explicitly notify you if markdown format is necessary. Secondly, when configuring the basic parameters of the simulated annealing algorithm, we set the initial temperature (T) to 2000, the stable temperature (T) to 10, and the inner loop iterations to 50. We compute the mechanical specific energy value using the current drilling parameters. Subsequently, we randomly adjust three drilling parameters - weight on bit (WOB), rotational speed, and flow rate – based on the current temperature, and update the drilling parameters accordingly. We input the updated drilling parameters into the specific energy model to compute the mechanical specific energy value under the current drilling parameter settings. If the current mechanical specific energy value is lower than the previously computed one, we keep the latest mechanical specific energy value. If the optimized result exceeds the computed mechanical specific energy value, there is still a probability of accepting the result. The inner loop continues iterating until it either reaches the specified number of iterations or fulfills the parameter setting conditions within the inner loop, at which point it exits, and the outer loop temperature is incremented. Upon stabilization of the outer loop temperature, the current parameters constitute the final optimized result.

The specific steps for optimizing drilling parameters are outlined below.

Set the initial temperature, and then acquire the Mechanical Specific Energy (MSE; denoted as M for brevity) and drilling parameters (p) using real-time drilling data.

(2) Inner loop: 1) Randomly select a parameter combination within the specified range, which includes drill pressure, rotational speed, and flow rate, and update the corresponding values in the real-time drilling parameters. Calculate the mechanical specific energy values M and M′ for parameters p and p′ using the model. If the mechanical specific energy value for parameter p is smaller than that for p′, update p to p′; otherwise, apply the Metropolis criterion: if $\text{exp}\left(-\frac{{\text{M}}^{{}^{\prime }}-\text{M}}{\text{t}}\right)>\text{random}(\text{0},\text{1})$, then update p to p′. 2) Repeat step 1) if the stopping condition for the inner loop (e. g., stability of the mean mechanical specific energy or reaching a predetermined number of iterations) has not been met.
(3) Outer Loop:Decrease the temperature; If the stopping criteria for the outer loop are not satisfied (e. g., reaching the minimum temperature, exceeding the maximum number of iterations, or the minimum mechanical specific energy value deviating from the optimization range).

### 5.3 Analysis of optimization results and engineering analysis

#### 5.3.1 Optimization results.

**Fig 22:**
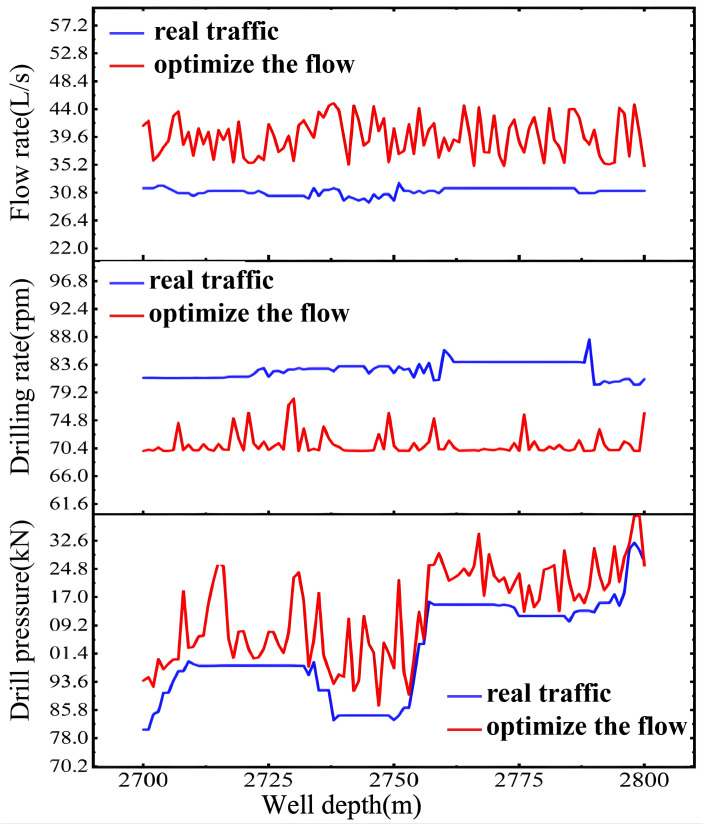
Optimization Results of Drilling Parameters.

Tests were conducted on the historical dataset of Well Zhanghai A, following the established optimization process for drilling parameters. Three adjustable drilling parameters—weight on bit, rotational speed, and flow rate—were taken into account. The variation range for each parameter was constrained based on different well sections. The results of optimizing the drilling parameters are depicted in Fig 22.

Based on the drilling parameter optimization results, the weight on bit and flow rate should be increased moderately, while the rotational speed should be reduced moderately. Prior to optimization, the average weight on bit was 102.19 kN, which increased to 119.96 kN post-optimization, indicating an approximate 17.39% increase. Before optimization, the average rotational speed was 82.76 rpm, and it decreased to 71.52 rpm after optimization, reflecting an approximate 13.58% decrease. Before optimization, the average inlet flow rate was 30. 97 L/s, which increased to 39.59 L/s post-optimization, showing an approximate 27.83% increase. The variations in weight on bit, rotational speed, and flow rate remained within the predefined limits, without notable fluctuations, aligning with petroleum engineering theories. This demonstrates the practical importance of the parameter optimization.

#### 5.3.2 Engineering analysis.

To assess the practical engineering efficacy of the parameter optimization, we chose Well Zhanghai A-1, a well adjacent to Well Zhanghai A, for our validation study. Based on our analysis of the formation lithology characteristics, we will conduct validation of the parameter optimization by concentrating on the 2700m-2800m interval in Well Zhanghai A-1.

(1) Lithological characteristics

The lithological characteristics of the formation in Well Zhanghai A-1 are detailed in Table 24.

**Table 24:** Lithology Table of Well Zhanghai A-1 Formation.

Stratification	Bottom boundary depth(m)	Layer thickness(m)	Lithological characteristics
Plain Formation	369	369	A suite of brownish-yellow silty clay interbedded with various sand layers of unequal grain size. It contains fossils such as spores and pollen, gastropods, and bivalves.
Minghuazhen Formation	1884	1515	Earthy yellow and brownish-red mudstone and sandy mudstone. The upper section exhibits coarser grain size and lighter color, containing iron-manganese nodules and calcareous concretions, while the lower section displays finer grain size and darker color.
Guantao Formation	2248	364	Grayish-white, grayish-green, and dark purplish-red sandstone and mudstone, interbedded with conglomeratic sandstone and conglomerate.
Dongying Formation	2690	442	Grayish-green and brownish-red mudstone interbedded with sandstone.
Member 1 of the Shahejie Formation	2840	150	Grayish-brown fluorescent marl and grayish-brown fluorescent calcareous mudstone.
Member 2 of the Shahejie Formation	3042	202	Light gray fluorescent fine-grained sandstone.
Member 3 of the Shahejie Formation	3230	188	Light gray fluorescent calcareous sandstone.

The lithological characteristics of the formations in the 2700m-2800m intervals of Well Zhanghai A and its adjacent well, Well Zhanghai A-1, are similar. Both intervals include the Guantao and Dongying formations. The Guantao Formation lithology comprises grayish-green to dark purplish-red sandstone and mudstone, interbedded with pebbly sandstone and conglomerate. The Dongying Formation lithology includes grayish-white, grayish-green, and dark purplish-red sandstone and mudstone, interbedded with pebbly sandstone and conglomerate.

(2) Sliding window selection

**Fig 23:**
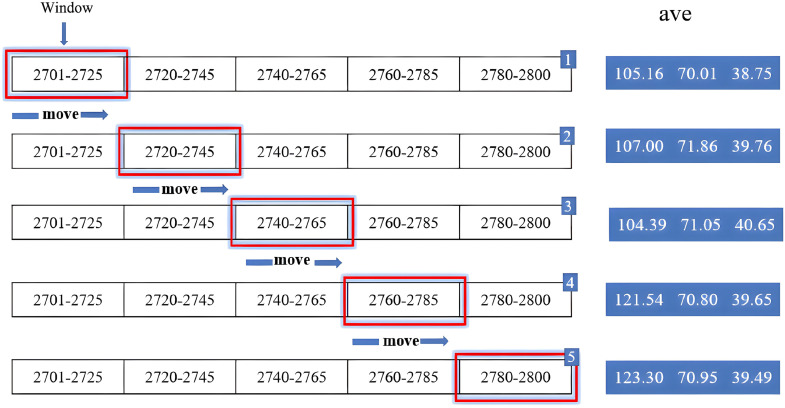
Illustration of the Sliding Window Concept.

In practical drilling engineering, drilling parameters often fluctuate significantly on a per-meter scale, making real-time adjustment difficult [47]. To address this issue, this study introduces the concept of sliding window segmentation to smooth and partition the optimized drilling parameters. The sliding window length is set to *k* = 25 m, with a step size of *h* = 20 m. As the window shifts forward, a 5 m overlap is maintained between consecutive windows to ensure parameter continuity and smooth transitions. The traversal process of the sliding window is illustrated in Fig 23.

Taking the well depth interval of 2700–2800 m as an example, the segmentation results obtained using the sliding window method are as Table 25:

**Table 25:** Sliding window quantitative data.

Window number	Depth intervals/m	Average drilling pressureWOB (kN)	Average speedRPM (r/min)	Average displacement Q (L/s)
1	2700–2720	94.7	81.56	29.87
2	2720–2745	102.3	81.43	29.87
3	2740–2765	108.6	81.38	29.90
4	2760–2785	118.2	81.33	29.92
5	2780–2800	122.7	81.30	29.95

As shown in the table, within the depth range of 2700–2765 m, the variation in weight on bit (WOB) is minimal, with a fluctuation range of approximately ±4.5%, indicating relatively stable formation characteristics. Beyond 2760 m, the WOB increases significantly by about 28%, suggesting a harder formation that requires a higher WOB to maintain the rate of penetration (ROP). Statistical analysis shows that after applying the sliding window method, the standard deviation of WOB decreases from 8.9 kN to 3.0 kN—a reduction of approximately 66%. Similarly, the fluctuation range of rotational speed decreases from ±0.4 r/min to ±0.15 r/min. The model-predicted ROP increases by an average of 16.1%, while the prediction error (MAE) decreases by about 23%.

These results demonstrate that the sliding window method effectively suppresses abrupt fluctuations caused by point-by-point optimization while maintaining parameter representativeness and stability. Consequently, the optimization results become more practical and applicable in real-world engineering operations.

(3) Application effect of adjacent wells

**Fig 24:**
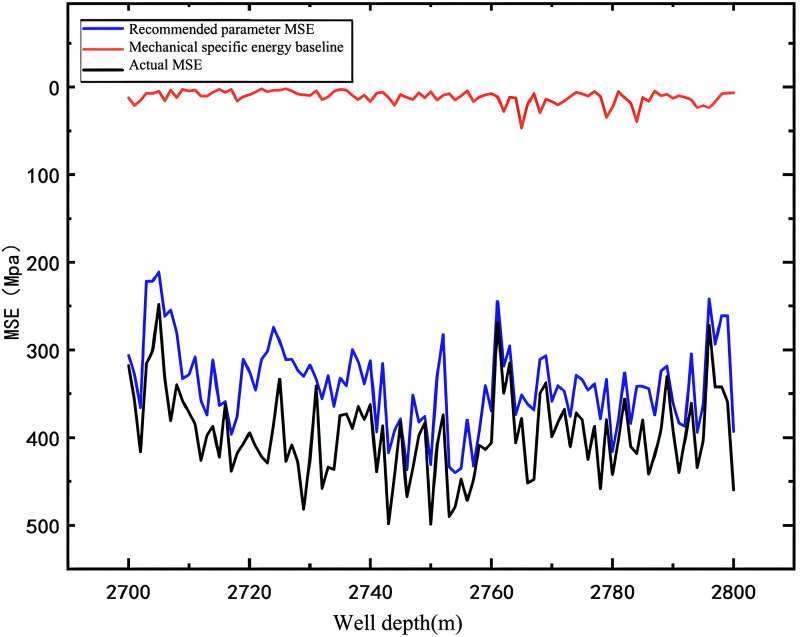
Application Effect Diagram of Adjacent Wells.

Empirical validation was performed on adjacent well Zhanghai A-1, with the optimization results depicted in Fig 24.

As illustrated in Fig 24, the red curve indicates the mechanical specific energy (MSE) baseline, representing the peak MSE value attainable during drilling to achieve the highest rock-breaking efficiency. The black curve illustrates the MSE profile prior to drilling parameter optimization, whereas the blue curve depicts the MSE profile post-optimization. It is evident from Fig 24 that the post-optimization MSE profile aligns more closely with the MSE baseline, suggesting that the MSE needed to fracture a unit volume of rock using the optimized drilling parameter set is lower than that required prior to optimization. This, in turn, confirms that the optimized drilling parameter set boosts rock-breaking efficiency.

(4) Transfer efficiency

Here, we introduce the transfer efficiency coefficient (EFF) for mechanical specific energy [47], defined as the measure of the drill bit’s rock-breaking efficiency.


$$EFF=\frac{CCS}{MSE}$$ (5-2)

Using the mechanical specific energy (MSE) values measured before and after optimization, along with the rock’s compressive strength (CCS), we calculated the drill bit’s rock-breaking efficiency for both the pre- and post-optimization scenarios.

**Fig 25:**
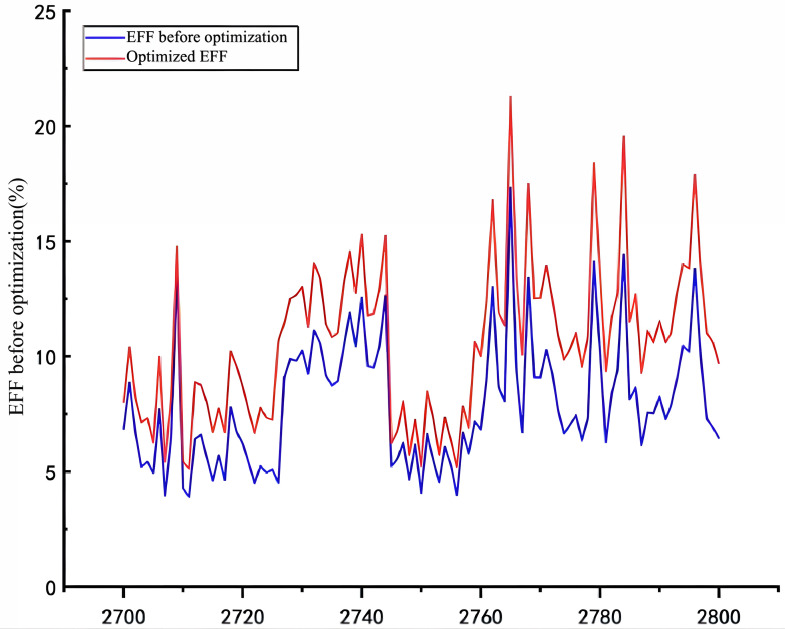
Comparison Chart of Transmission Efficiency.

As shown in Fig 25, the optimized drilling parameter set significantly boosts the transfer efficiency of mechanical specific energy. This leads to an average 43. 34% improvement in rock-breaking efficiency, subsequently enhancing drilling efficiency, shortening the oil production cycle, and cutting development costs. Consequently, it provides robust guidance for practical engineering applications.

## 6. Conclusion and outlook

### 6.1 Conclusion

In this study, an intelligent drilling parameter optimization method based on a hydraulic–mechanical specific energy model is proposed, and its effectiveness is validated using field data. The main conclusions are as follows:

(1) The developed hybrid data preprocessing workflow significantly enhances data quality and boosts the signal-to-noise ratio. It efficiently eliminates “data burrs” without compromising the authentic underlying trends, thus establishing a dependable basis for model training.
(2) The proposed hydraulic–mechanical specific energy model comprehensively accounts for the effects of hydraulic parameters on rock-breaking efficiency. The BP neural network achieved an accuracy of R² = 0. 9 in ROP prediction, which is significantly higher than that of the traditional method (R² = 0. 7).
(3) Parameter sensitivity analysis indicates that weight on bit (WOB), rotational speed, and flow rate are the three key factors influencing MSE. Among them, WOB has the most significant effect, followed by rotational speed and flow rate.
(4) Based on the optimization scheme combining the simulated annealing algorithm with the sliding window approach, field validation across four wells demonstrated an average improvement in drilling efficiency of 43. 34%, confirming the strong engineering applicability and practical value of the proposed method.

### 6.2 Outlook

The optimization method based on the hydraulic–mechanical specific energy model proposed in this study provides an effective solution for achieving intelligent drilling. Building upon the current research outcomes, future work can be directed toward the following areas:

(1) Development of a multi-objective optimization system: The current model focuses on mechanical specific energy as the primary optimization objective. Future efforts will aim to establish a multi-objective optimization framework that comprehensively considers drilling efficiency, operational safety, and economic performance. A collaborative optimization model integrating mechanical specific energy, wellbore stability, and cost control will be developed to achieve balanced performance across multiple dimensions.
(2) Exploration of Diverse Intelligent Optimization Algorithms: At present, the simulated annealing algorithm is the sole method utilized for optimizing drilling parameters, whereas intelligent optimization algorithms continue to evolve rapidly. Looking ahead, it is recommended to incorporate more sophisticated optimization algorithms, including genetic algorithms [48], particle swarm optimization [49], and ant colony algorithms [50]. Furthermore, the selection of optimal algorithm combinations should be tailored to different geological formations and operational scenarios to improve the optimization’s efficacy and resilience.
(3) Promotion of real-time engineering applications: The optimization system will be deeply integrated with digital twin platforms to develop a real-time drilling guidance system with online learning capabilities. This integration will enable dynamic optimization and risk prediction of drilling parameters, advancing drilling operations toward comprehensive intelligence and automation.
(4) Expansion of stratigraphic adaptability: Further validation and optimization of the model will be conducted in complex formations (e. g., salt–gypsum layers, igneous rocks, etc.). A cross-regional knowledge base for parameter optimization will be established to enhance the generalizability and applicability of the proposed method across diverse geological settings.

## Supporting information

S1 Appendix Symbol Table. (DOCX)
