# Supplementary material for: Research on an intelligent drilling parameter optimization method using sliding window segmentation based on the hydraulic-mechanical specific energy model
Source: PLoS One. 2026 Jan 2;21(1):e0339324. doi: 10.1371/journal.pone.0339324 (PMC12758761; doi:10.1371/journal.pone.0339324)
Supplement: S1 Appendix — (DOCX) [file pone.0339324.s001.docx]

**Symbol Table**

| Symbol | Unit | Definition |
| --- | --- | --- |
| MSE | kPa | Mechanical Specific Energy; the energy required to fracture a unit volume of rock. |
| S-G | – | Savitzky-Golay filter; a low-pass filtering method based on the least squares principle, used for data smoothing to retain data trends while reducing high-frequency noise. |
| LOF | – | Local Outlier Factor; a density-based anomaly detection method that identifies outliers by comparing the local reachability density of a data point with that of its neighbors. |
| ROP | m/h | Rate of Penetration; the depth drilled by the bit per unit time, a key indicator of drilling efficiency. |
| BP | – | Backpropagation Neural Network; an algorithm involving forward signal propagation and backward error propagation, used to adjust network weights for improved prediction accuracy. |
| WOB | kN | Weight on Bit; the force applied to the drill bit, a key parameter affecting drilling efficiency and mechanical specific energy. |
| UDC | – | Unit Drilling Cost; the cost incurred per unit depth drilled, an important metric for evaluating drilling economic performance. |
| RF | – | Random Forest; an ensemble learning algorithm capable of capturing complex relationships and nonlinear patterns in data, used for imputing missing values in drilling data. |
| CCS | – | Compressive Strength of Rocks; the ability of rock to resist failure under uniaxial compression, used for calculating the mechanical specific energy baseline. |
| RMSE | – | Root Mean Square Error; a metric for the average deviation between model predicted values and actual values, calculated as the square root of the average of squared differences. |
| MAPE | % | Mean Absolute Percentage Error; evaluates prediction accuracy by calculating the average of the absolute percentage deviations between predicted and actual values. |
| R² | – | Coefficient of Determination; measures the proportion of variance in the data explained by the model, ranging from 0 to 1, with values closer to 1 indicating better fit. |
| MWD | – | Measurement While Drilling; a technology for real-time downhole parameter measurement (e.g., torque, pressure) during drilling and transmission to the surface. |
| EFF | % | Transfer Efficiency Coefficient; measures the rock-breaking efficiency of the drill bit, reflecting the conversion effectiveness of mechanical energy into rock-breaking work. |
| T | kN·m | Torque; the rotational moment generated by the drill string during drilling, an important parameter for calculating mechanical specific energy. |
| RPM | r/min | Rotational Speed; the number of rotations of the drill bit per minute, a key adjustable parameter affecting drilling efficiency and mechanical specific energy. |
| Q | L/s | Flow Rate; the volumetric flow rate of drilling fluid through the bit nozzles, affecting bottomhole cleaning and hydraulic rock breaking. |
| d_B_ | m | Bit Diameter; the nominal diameter of the drill bit, used for calculating bottomhole area and volume of rock fractured. |
| Δt | min | Drilling Time; the time interval under a specific set of drilling parameters, used for calculating work done by the drill string and volume of rock fractured. |
| AB | m² | Bottom-hole Area; the contact area between the drill bit and the bottomhole rock, calculated from the bit diameter. |
| MSE_h_ | kPa | Hydraulic-Mechanical Specific Energy; a mechanical specific energy model incorporating the effects of hydraulic parameters, combining mechanical and hydraulic energy contributions to rock breaking. |
| MSE_input_ | – | Total Input Energy per Unit Area; the total energy input per unit area of the bottomhole, including both mechanical and hydraulic energy. |
| ROP_output_ | m/h | Output Rate of Penetration; the actual rate of penetration considering hydraulic effects. |
| W_total_ | kJ/min | Total Work per Unit Time; the total work done by all forces (WOB, torque, hydraulic) for rock breaking per unit time. |
| V_ROP_ | m³/min | Rock Volume Excavated per Unit Time; the volume of rock fractured by the drill bit per unit time, calculated from the ROP and bottomhole area. |
| W_WOB_ | kJ/min | Work per Unit Time by WOB; the work done by the weight on bit on the formation per unit time. |
| W_RPM_ | kJ/min | Work per Unit Time by Torque; the work done by the bit torque on the formation per unit time. |
| W_HJ_ | kJ/min | Work per Unit Time by Fluid Jet; the work done by the drilling fluid jet on the formation per unit time. |
| WOBe | kN | Effective Weight on Bit; the actual effective force applied to the bit after accounting for the reaction force of the nozzle jet, calculated as WOBe = WOB - ηFj. |
| η | – | Energy Reduction Coefficient; accounts for energy loss of the drilling fluid from the nozzle exit to the bottomhole, typically ranging from 25% to 40%. |
| F_j_ | kN | Jet Impact Force at Nozzle Exit; the impact force exerted by the drilling fluid jet on the formation as it exits the nozzle, generating a reaction force that affects effective WOB. |
| ρ_d_ | g/cm³ | Drilling Fluid Density; the mass per unit volume of drilling fluid, affecting pressure transmission and wellbore stability. |
| A_0_ | mm² | Total Cross-Sectional Area at Nozzle Exits; the sum of the cross-sectional areas of all bit nozzle exits, influencing drilling fluid flow rate and jet impact force. |
| d_i_ | mm | Diameter of the i-th Nozzle; the nominal diameter of a single nozzle, used for calculating nozzle exit cross-sectional area. |
| z | – | Number of Nozzles; the total number of nozzles installed on the drill bit. |
| HP | kW | Hydraulic Power at Drill Bit; the effective hydraulic power acting on the bottomhole, reflecting the contribution of hydraulic energy to rock breaking. |
| ΔP_b_ | MPa | Pressure Drop across the Bit; the pressure loss as drilling fluid flows through the bit, used for calculating bit hydraulic power and jet impact force. |
| V_op_ | m/h | Predicted Mechanical Drilling Rate; the predicted ROP calculated using the ternary drilling rate equation. |
| C | – | Comprehensive Coefficient; an empirical coefficient in the ternary drilling rate equation, related to factors such as bit type and formation characteristics. |
| Kf | – | Drillability Index; an indicator of the ease with which formation rock can be fractured by the drill bit, comprehensively reflecting the influence of rock physico-mechanical properties on drilling. |
| α | – | Weight-on-bit Exponent; the exponent for the influence of WOB on ROP in the ternary drilling rate equation, reflecting the sensitivity of ROP to changes in WOB. |
| λ | – | Rotational-speed Exponent; the exponent for the influence of rotational speed on ROP in the ternary drilling rate equation, reflecting the sensitivity of ROP to changes in RPM. |
| γ | – | Formation-drillability Exponent; the exponent for the influence of formation drillability on ROP in the ternary drilling rate equation, reflecting the constraint of formation characteristics on ROP. |
| Rop | min/m | Drilling Time per Meter; the time required for the drill bit to drill one meter of formation, as recorded in logging data, used for calculating the formation drillability index. |
| E | – | Model Computational Accuracy; a metric for the accuracy of the ROP prediction model, calculated from the deviation between actual and predicted ROP. |
| μ | – | Sliding Friction Coefficient of the Bit; the coefficient of sliding friction between the drill bit and the bottomhole rock, used for calculating bit torque. |
| ρ | m | Distance from a Point to the Center; the distance from a micro-unit area to the center of the bit, used in the integral calculation of bit torque. |
| AC | μs/m | Acoustic Slowness; the time required for an acoustic wave to travel a unit distance through rock, used for calculating compressional wave velocity. |
| V_P_ | km/s | P-wave Velocity; the velocity of compressional waves propagating through rock, reflecting the compactness and elastic properties of the rock. |
| Δt_p_ | ms/m | P-wave Slowness; the time difference for a P-wave to travel a unit distance, inversely related to P-wave velocity. |
| V_s_ | km/s | Shear-wave Velocity; the velocity of shear waves propagating through rock, used for calculating the elastic modulus of rock. |
| Δt_s_ | ms/m | Shear-wave Slowness; the time difference for an S-wave to travel a unit distance, inversely related to S-wave velocity. |
| E | GPa | Young's Modulus of Elasticity; the ratio of stress to strain in the elastic deformation stage of rock, reflecting its resistance to elastic deformation. |
| ρ | g/cm³ | Density; the mass per unit volume of rock, used for calculating compressive strength and elastic modulus. |
| k | – | Length of Sliding Window; the number of data groups contained in the sliding window for segmented drilling parameter optimization, set to 25 in this study. |
| h | – | Sliding Step Size; the number of data groups the sliding window moves right each time, set to 20 in this study. |
| Standpipe Pressure | MPa | The pressure of the drilling fluid measured at the surface standpipe, reflecting the pressure status of the drilling fluid circulation system. |
| Hook Load | kN | The total weight borne by the hook (including drill string, bit, etc.), reflecting the load condition of the downhole assembly. |
| Annular Pressure Loss | MPa | The pressure loss of drilling fluid flowing through the annular channel between the drill string and the wellbore wall, affecting bottomhole pressure and drilling fluid circulation efficiency. |
| Guantao Formation | – | A formation name, one of the important strata in the study area (Zhanghai Oilfield), primarily characterized by grayish-white, grayish-green, and dark purplish-red sandstone and mudstone. |
| Dongying Formation | – | A formation name, a key stratum in the study area, characterized by interbedded grayish-green and brownish-red mudstone and sandstone. |
| Shahejie Formation | – | A formation name, a deep stratum in the study area, divided into Members 1, 2, and 3, with lithology including grayish-brown fluorescent marl, fine sandstone, and calcareous sandstone. |
| Minghuazhen Formation | – | A formation name, primarily consisting of earthy yellow and brownish-red mudstone and sandy mudstone; the upper section has coarser grains and lighter color, containing iron-manganese nodules and calcareous concretions. |
| Pingyuan Formation | – | A formation name, characterized by a suite of interbedded brownish-yellow silty clay and sand layers of varying grain sizes, containing fossils such as spores, pollen, gastropods, and bivalves. |
